# VentX promotes tumor specific immunity and efficacy of immune checkpoint inhibitors

**DOI:** 10.1016/j.isci.2023.108731

**Published:** 2023-12-14

**Authors:** Yi Le, Hong Gao, Joanna Le, Jason L. Hornick, Ronald Bleday, Jon Wee, Zhenglun Zhu

**Affiliations:** 1 Department of Medicine, Brigham and Women's Hospital, Boston, MA, USA; 2 Department of Medicine, Tufts Medical Center, Boston, MA, USA; 3 Department of Surgery, Brigham and Women's Hospital, Boston, MA, USA; 4 Department of Pathology, Brigham and Women’s Hospital, Boston, MA, USA; 5 Department of Obstetrics and Gynecology, University of Massachusetts Medical Center

**Keywords:** Molecular biology, Immunology, Cancer

## Abstract

Immune suppression within tumor microenvironments (TME) have been implicated in limited efficacy of immune check point inhibitors (ICIs) against solid tumors. Down-regulated VentX expression in tumor associated macrophages (TAMs) underlies phagocytotic anergic phenotype of TAMs, which govern immunological state of TME. In this study, using a tumor immune microenvironment enabling model system (TIME-EMS) of non-small cell lung cancer (NSCLC), we found that PD-1 antibody modestly activates cytotoxic T lymphocytes (CTLs) within the NSCLC-TME but not the status of TIME. We showed that the restoration of VentX expression in TAMs reignites the phagocytotic function of TAMs, which in turn, transforms TIME, activates CTLs in a tumor-specific manner and promotes efficacy of PD-1 antibody against NSCLC but not toxicity on normal lung epithelial cells. Supported by *in vivo* data on NSG-PDX models of primary human NSCLC, our study revealed potential venues to promote the efficacy of ICI against solid tumors through VentX-based mechanisms.

## Highlights

• A TIME-EMS model to explore the function of ICIs in the context of TME
• Tumor specific activation of CTLs through VentX-based mechanism
• Methods to promote efficacy but not the toxicity of ICIs against solid tumor
• NSG-PDX models of primary human cancer for combined immunotherapy

## Graphical abstract

**Figure:**
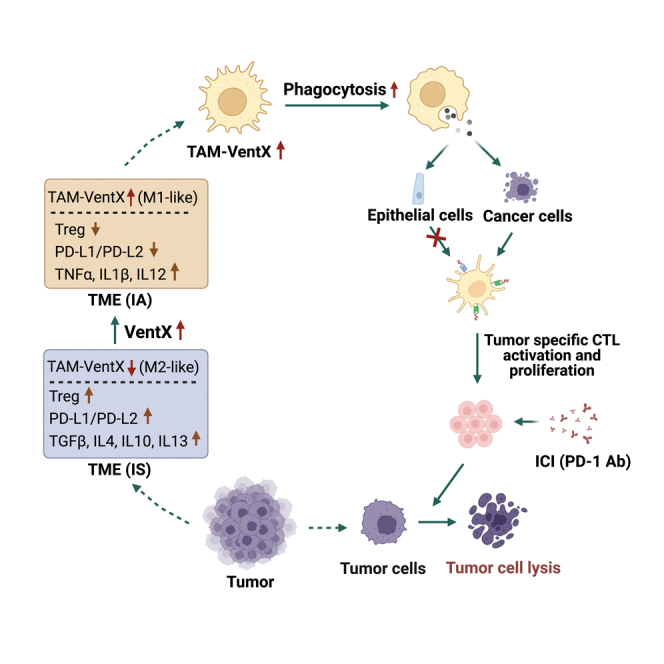

## Introduction

CD8^+^ cytotoxic T lymphocytes (CTL) are key immune effector cells in tumor immunology. Activated by T cell receptor (TCR) engagement with peptide antigens presented on major histocompatibility complex (MHC), CD8 T cell functions are modulated by co-stimulatory and co-inhibitory receptors to ensure balance between the immune reaction to foreign/tumor antigens and excessive auto-immune inflammatory response.^1^^,^^2^ During tumorigenesis, the interaction of co-inhibitory checkpoint receptors with their ligands on tumor and stromal cells accounts for the inhibition/exhaustion of tumor-associated CD8 T cells and tumor cells’ avoidance of immune destruction.^3^^,^^4^ Immune checkpoint inhibitors (ICIs), such as PD-1 and CTLA-4 antibodies, function in part by blocking CD8 inhibition, thereby reinvigorating CD8^+^ CTL function.^5^ While the clinical application of ICIs has achieved unprecedented success in treating selected types of cancers, especially hematological malignancies, the efficacy of ICIs against solid tumors remains limited. For non-small cell lung cancer (NSCLC) for example, only 20% of patients respond to ICIs, moreover, the majority of the responders eventually relapse and succumb to the disease during 5-year follow-up.^6^^,^^7^^,^^8^

Inadequate antigen presentation and immune suppression of CD8^+^ CTLs within the tumor microenvironment (TME) have been proposed as two possible reasons for limited efficacy of ICIs against solid tumors.^9^ Nonetheless, the potential clinical benefit of ICI combination therapies with neoantigen stimulation or presentation, for example with antibodies to block CD47 “the do not eat me” signal, have yet be demonstrated in clinical trials.^10^^,^^11^^,^^12^ Likewise, numerous approaches have been developed to address immune suppression within the TME as a potential approach to improve efficacy of cancer immunotherapy. However, with the lack of a practical platform to dissect the functional interaction of ICIs and immune cells within the TME, the potential benefit of targeting TME in the treatment of solid tumors remains a hypothesis to be tested in the early phases of clinical trials.^13^^,^^14^ In addition to therapeutic resistance, immune related adverse effects (irAE) induced by ICI treatment presents another major challenge that confronts the clinical application of ICI in cancer treatment.^15^

The human homeobox protein VentX is a human homologue of the vertebrate *Xenopus* ventral homeobox cell fate determination gene Xom, a lymphocyte enhancing factor/T cell factor (LEF/TCF)-associated transcription factor that antagonizes the dorsal Wnt/beta-catenin signaling during early *Xenopus* embryogenesis.^16^^,^^17^^,^^18^^,^^19^ Initially identified through the reverse genetic modeling of dorsoventral axis formation during early vertebrate embryogenesis,^18^^,^^19^ recent translational and mechanistic study showed that VentX plays a key role in controlling the proliferation and differentiation of human hematopoietic cells during blood ontogenesis and leukemogenesis through modulating the cell cycle machinery and signaling pathways involved in the process.^19^^,^^20^^,^^21^ In addition, it was found that VentX governs the phagocytosis and plasticity of mononuclear phagocytes by modulating signaling, such as SHP-1, SHP-2, M-CSFR, TLR and IFNγ.^20^^,^^22^ More recently, we characterized immunological state of primary human samples of colorectal cancer (CRC) and pancreatic ductal adenocarcinoma (PDAC) and found that down-regulated VentX expression in tumor associated macrophages (TAMs) underlies the immune suppression of CRC and PDAC TME.^22^^,^^23^ Using *en bloc* tumor and macrophage co-culture models, we found that VentX-modulated-TAMs govern the immunological state of tumor immune microenvironment (TIME) by modulating the differentiation and proliferation of tumor infiltrating T lymphocytes (TILs).^24^ We showed further that the restoration of VentX expression in TAMs exerts strong inhibition on tumorigenesis in pre-clinical NSG-PDX models of primary human CRC and PDAC.^22^^,^^23^^,^^24^

In the current study, we characterized the immune cell composition of NSCLC-TME, which exhibits an immune suppressive landscape with abundant pro-tumor M2-like TAMs, CD8 T cells with exhausted phenotype and increased number of immune regulatory Treg cells. We found that VentX expression is significantly down-regulated in the NSCLC-TAMs. Using a tumor TIME-enabling model system (TIME-EMS),^24^ we analyzed the functional interaction of ICI and immune cells *ex vivo* in the context of NSCLC-TME. We found that the application of PD-1 antibody to the TIME-EMS of NSCLC led to the modest activation of CD8 T cells but no significant alteration in the immune landscape of NSCLC-TME. We showed that the restoration of VentX expression in NSCLC-TAMs reverts immune landscape of NSCLC-TME and promotes the tumoricidal effects of PD-1 antibody on NSCLC cells about 4-fold but does not alter its cytocidal effects on normal lung epithelial cells. On a more mechanistic level, we showed that the restoration of VentX expression in TAMs promotes TAM phagocytosis, which in turn promotes the tumor-specific activation of CD8 T cells. Consistent with these *ex vivo* findings, we showed that VentX-modulated-TAMs enhance the efficacy of PD-1-antibody against NSCLC tumorigenesis *in vivo* in pre-clinical NSG-PDX models of primary human NSCLC. Taken together, our findings suggested that TIME-EMS can serve as an effective *ex vivo* platform to explore ICI function under the physiological context of TME and potential approaches of VentX-modified-TAMs in promoting the tumor-specific activation of CTLs and efficacy of ICIs against solid tumors, such as NSCLC.

## Results

### Characterization of immune cells in non-small cell lung cancer-tumor microenvironments

**Figure 1:**
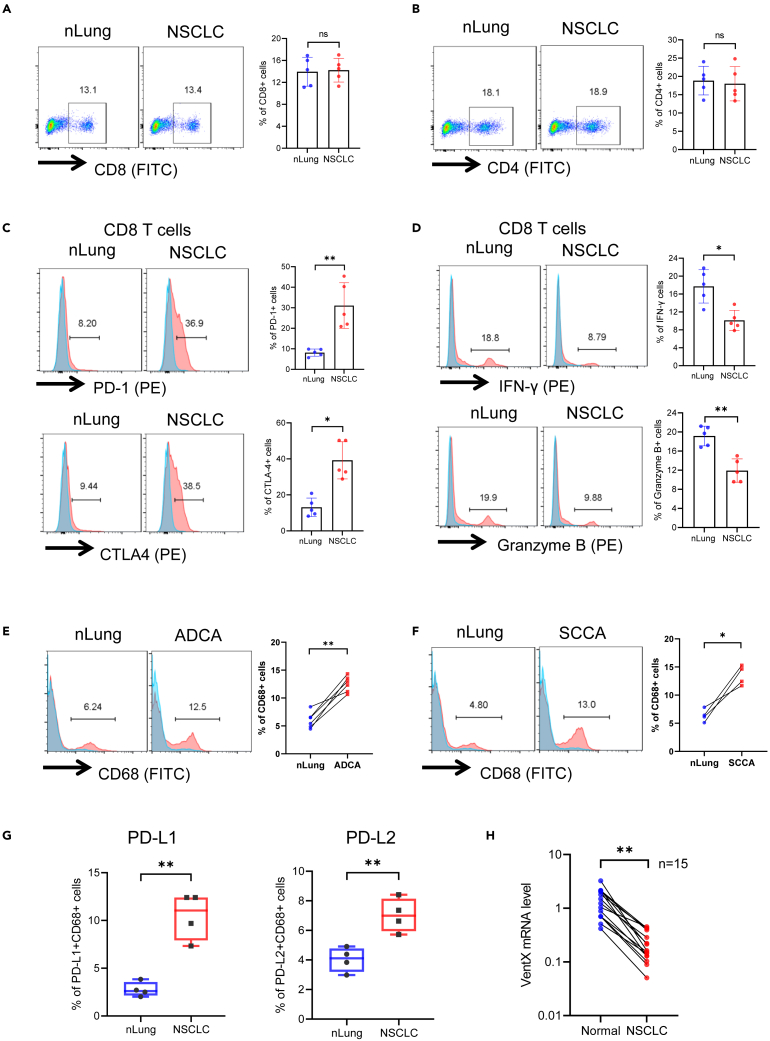
Profiling of Immune cell compositions of NSCLC (A and B) FACS analysis of CD8^+^ (A) and CD4^+^ (B) T cells in NSCLC and nLung tissues, n = 5, the ns stands for no statistically significant difference. (C) Characterization of CD8 cells isolated from nLung tissues and NSCLC. (C) CD8 T cells isolated from NSCLC and nLung tissues were stained with fluorescence conjugated PD-1 and CTLA4 antibodies and then subjected to FACS analysis. (D) Isolated CD8 T cells were stimulated with Dynabeads for 48 h at 37°C and then subjected to FACS analysis of intracellular IFNγ and anti-Granzyme B expression. Data represent means ± S.D. of five independent experiments, n = 5, ∗p < 0.05, ∗∗p < 0.01 by paired Student’s t test. (E and F) Paired comparison of CD68^+^ macrophages in nLung verses L-ADCA (E) and L-SCCA (F). CD68^+^ macrophages in nLung tissue and L-ADCA and L-SCCA were analyzed by a flow cytometer. Blue shaded peaks were isotype control, red shaded peaks were CD68^+^ cells, n = 5. Statistical significance was defined as ∗∗p < 0.01, calculated by paired Student’s *t* test. (G) Paired comparison of cell surface expression of PD-L1 and PD-L2 on macrophages isolated from nLung and NSCLC by FACS analysis. Results represent mean ± SD of four independent experiments. ∗∗ indicates p < 0.01 by paired Student’s t test. (H) Paired comparison of VentX mRNA expression in macrophages isolated from nLung and NSCLC of 15 patients by qRT-PCR analysis. The relative VentX mRNA expression levels in nLung macrophages were arbitrarily designated as 1. ∗∗ indicates p < 0.01 TAMs vs. control macrophages isolated from nLung, n = 15 by paired Student’s *t* test.

About 85% of lung cancers are NSCLC, of which about 70% are adenocarcinoma (L-ADCA) and 20% are squamous cell carcinoma (L-SCCA).^25^ While the abundance and diversity of immune cells in NSCLE-TME has been appreciated, the mechanisms underlying ICI resistance in rescuing CD8 exhaustion at NSCLC-TME have yet to be elucidated.^9^ Using freshly isolated tissues, we carried out a systemic evaluation of immune cells in paired NSCLC and control non-involved lung tissues (nLung) of patients with NSCLC with FACS analysis (Figures S1A–S1D). Consistent with prior findings, there was no significant reduction of the numbers of CD8^+^ and CD4 + lymphocytes in NSCLC (Figures 1A and 1B). Nevertheless, the NSCLC CD8 T cells demonstrated an immune exhaustion phenotype with the elevated expression of cell surface checkpoint inhibitors PD-1 and CTLA-4 and decreased the expression of effector molecules, such as IFN-γ and Granzyme B, upon stimulation (Figures 1C and 1D). Consistent with prior findings,^9^^,^^26^ we showed that NSCLC contained more Treg cells than did control nLung tissues (Figures S1E and S1F). TAMs are key executors of both innate and adaptive immunity in the TME. Unlike immune “cold” tumors, such as the pancreatic cancer, NSCLC is considered to be an immune “hot” tumor that contains abundant immune cells.^27^ Moreover, chronic inflammation has been implicated in the pathogenesis of NSCLC.^8^^,^^28^^,^^29^ As tissue cues exert a significant impact on the phenotype and function of macrophages,^30^ we further characterized the distribution and phenotype of NSCLC-TAMs. We found that, in comparison with the control nLung tissues, both L-ADCA and L-SCCA contain significantly more macrophages (Figures 1E and 1F). We showed that these NSCLC-TAMs display a characteristic immune-suppressive M2-like phenotype with the increased expression of M2 markers and immune regulatory cytokines (Figure S2). Moreover, these TAMs exhibit more ICI ligands PD-L1 and PD-L2 on their surface (Figure 1G). Consistent with the role of VentX as a central regulator of NSCLC-TAM plasticity, our study showed that VentX expression in TAMs is significantly down regulated in all tested cases of NSCLC (Figures 1H).

### PD-1 antibodies activate cytotoxic CD8 T cells in non-small cell lung cancer-tumor microenvironments

**Figure 2:**
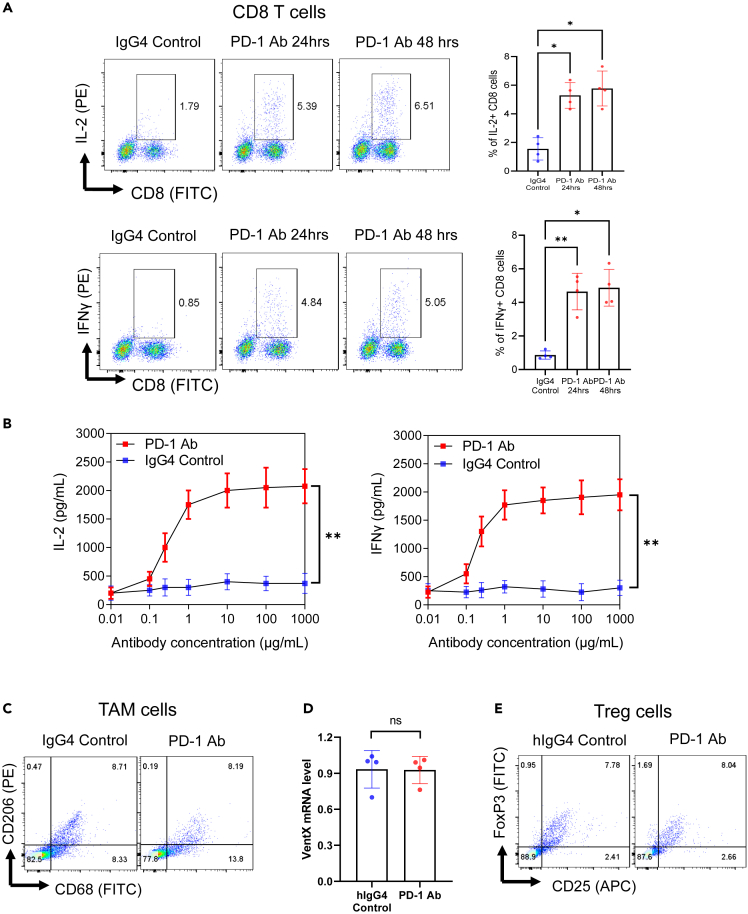
Effects of PD-1 antibody on immune cells in NSCLC-TME (A) PD-1 antibody activates CD8 T cells in NSCLC-TME. The *en bloc* NSCLC or nLung tissues were cultured in 24-well plates and treated with 1 μg/mL Pembrolizumab or human IgG4 control for 24 or 48 h. Single cell suspensions were then generated and stained with FITC conjugated anti-CD8 antibody. The cells were then fixed, permeabilized, stained with PE-conjugated anti-IL2, or anti-IFNγ antibodies and analyzed by a flow cytometry. Representative figures of three independent experiments were shown. Data represent means ± S.D. of three independent experiments, n = 3, ∗ p < 0.05, ∗∗ p < 0.01 by paired Student's t test. (B) Dosage-dependent stimulation of pro-inflammatory cytokine secretion by PD-1 antibody. The *en bloc* NSCLC and nLung tissues culture described above were treated with Pembrolizumab or human IgG4 control at concentration indicated. The culture media were collected after 48 h. The levels of IL-2, and IFN-γ were quantified using ELISA kits, n = 5. Statistical significance was determined by a two-way ANOVA analysis, ∗∗ indicates p < 0.01, Pembrolizumab vs. IgG4 control. (C and D) Effects of PD-1 antibody on TAMs in NSCLC-TME. The *en bloc* NSCLC or nLung tissues were treated with 1 μg/mL Pembrolizumab or human IgG4 and single cell suspensions were obtained as described above after 48 h. The effects of the treatment on TAMs were determined by FACS analysis of cell surface expression of CD68 and CD206 (C) and qRT-PCR analysis of VentX expression levels (D). Representative figure of four independent experiments were shown, the ns stands for no statistically significant difference. (E) Effects of PD-1 antibody on Treg in NSCLC-TME. The percentage of Treg cells in *en bloc* culture after Pembrolizumab or human IgG4 treatment described above were determined by FACS analysis of the percentage changes of the CD4^+^CD25+FoxP3+ cells. Representative figures of four independent experiments were shown. No significant changes were noticed.

The importance of TME in anti-cancer drug development has been increasingly appreciated,^31^ and targeting the TME has been suggested for combination therapy to boost ICI efficacy against NSCLC. However, in the absence of an *in vitro* platform to address ICI function in the context of TME, the efficacy of ICI-conjugated combination therapy can only be evaluated in pre-clinical animal models and early phase clinical trials. On the basis of our recent success in employing *en bloc* tumor culture to evaluate functional interaction between TAMs and TILs in the TME and the function of the tumor immune microenvironment enabling model system (TIME-EMS) to explore the mechanisms of chemo-immune interaction,^22^^,^^23^^,^^24^ we tested the potential application of *en bloc* cultures of NSCLC to evaluate the function of PD-1 antibodies in the TME. Small pieces of freshly isolated NSCLC or nLung tissues were incubated in RPMI-based media and subjected to PD-1 antibody treatment for indicated times (Figure 2). Tumor-infiltrating T cells and TAMs were then isolated and their functional status was determined by FACS analysis. We found that PD-1 antibody treatment activated CD8 T cells in TME, as revealed by the elevated expression and secretion of IL-2 and IFN-γ (Figure 2A). Consistent with the physiological role of PD-1 antibody in CD8 activation, we found that the effect of PD-1 antibody on CD8 T cell activation is dosage dependent (Figure 2B). Different from other *in vitro* assays, the function of TME in the PD-1 antibody activation of CD8 T cells was indicated by the findings that the application of PD-1 antibody led to the direct activation of CD8 T cells in TME, without the need for additional antigen or cytokine stimulation.^32^^,^^33^^,^^34^ These findings suggest that the employment of *en bloc* NSCLC culture provides an opportunity for the functional evaluation of ICIs under the physiological context of TME. The specificity of the assay platform was further indicated by our findings that the application of PD-1 antibody to the *en bloc* NSCLC culture caused no significant phenotypic changes in the Treg cells and TAMs. (Figures 2C–2E).

### VentX-regulated-tumor associated macrophages convert immune landscape of non-small cell lung cancer and promote the PD-1 antibody reinvigoration of CD8 T cells in tumor microenvironments

**Figure 3:**
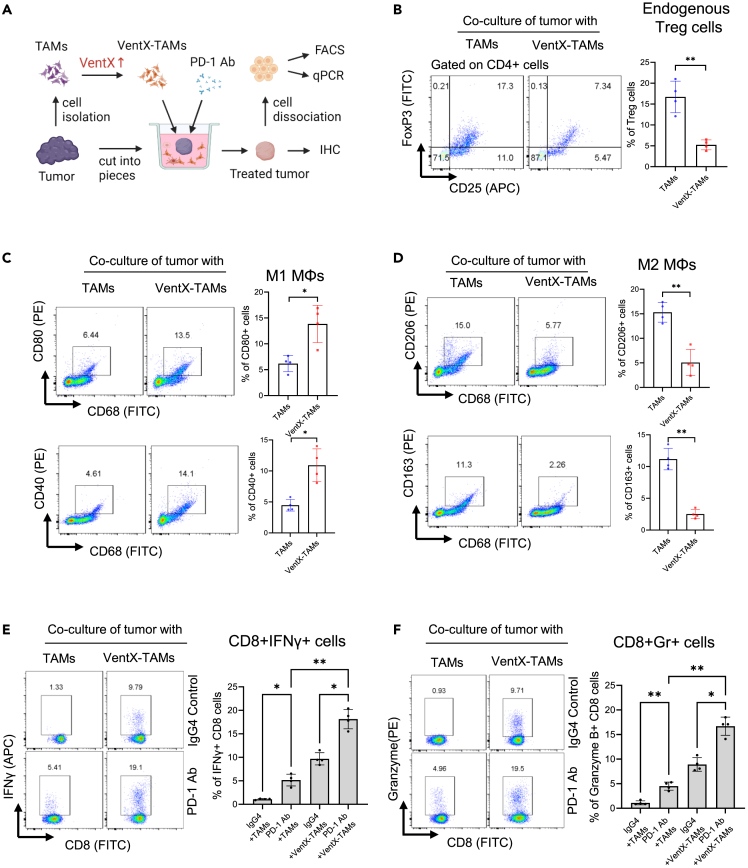
VentX-TAMs promote the PD-1 antibody activation of CD8 T cells in NSCLC-TME (A) Schematic diagram of experimental procedures for the treatment of *en bloc* tumor tissue with VentX-TAMs and PD-1 antibody. *En bloc* NSCLC tissues were incubated with autologous TAMs transfected with GFP-VentX or GFP control and PD-1 antibody for up to 5-day. Single cell suspensions were then generated by mechanical disruption, the tumor associated immune cells were analyzed by FACS analysis. (B) The effects of VentX-TAMs on Treg differentiation in NSCLC-TME as determined by FACS analysis of percentage of CD4^+^CD25+Foxp3+ cells (C and D) The effects of VentX-TAMs on endogenous TAMs in NSCLC-TME as determined by FACS analysis of percentage of indicated M1 and M2 cell surface markers. (E and F) Effects of VentX-TAMs on the PD-1 antibody activation of CD8 T cells as determined by FACS analysis using APC-conjugated anti-IFNγ (E) or PE-conjugated anti-Granzyme B antibodies (F). Data represent the mean ± SD of 4 independent experiments, ∗∗ indicates p < 0.01 by paired Student’s *t* test.

Using CRC and PDA as models, our recent studies showed that VentX modulates the plasticity of TAMs, which in turn reprograms the immune landscape of TME by dictating the differentiation of TILs.^22^^,^^23^ However, unlike CRC and PDA, NSCLC contains a unique TME with abundant immune cells and inflammatory cytokines.^9^^,^^35^ As TAM differentiation is modulated by environmental cues, and the effects of VentX on monocyte differentiation are modulated by extra-cellular signaling,^20^^,^^30^^,^^36^ the unique properties of NSCLC-TME prompted us to examine the effects of VentX on NSCLC-TAM phenotypes. We found that the restoration of VentX expression in TAMs promotes the polarization of NSCLC-TAMs from the pro-tumor M2-like phenotype to a more anti-tumor M1-like phenotypes (Figures S3A–S3H). Further, we found that, similar to CRC and PDA, VentX-regulated-TAMs (VentX-TAMs) re-program the immune landscape of NSCLC-TME from suppression to activation by modulating the differentiation of NSCLC-TAMs and CD4 T cells (Figures 3A–3D). As M2-TAMs and Tregs have been implicated in CD8 exhaustion in the TME, we asked whether VentX-TAMs enhance the PD-1 antibody reinvigoration of CD8 T cells in TME. As shown in Figures 3E and 3F, we found that the application of VentX-TAMs to the *en bloc* NSCLC culture led to about the 4-fold amplification of PD-1 antibody-induced CD8 T cell activation.

### VentX-regulated-tumor associated macrophages promote efficacy of PD-1 antibodies against non-small cell lung cancer through the tumor-specific activation of cytotoxic T lymphocytes

**Figure 4:**
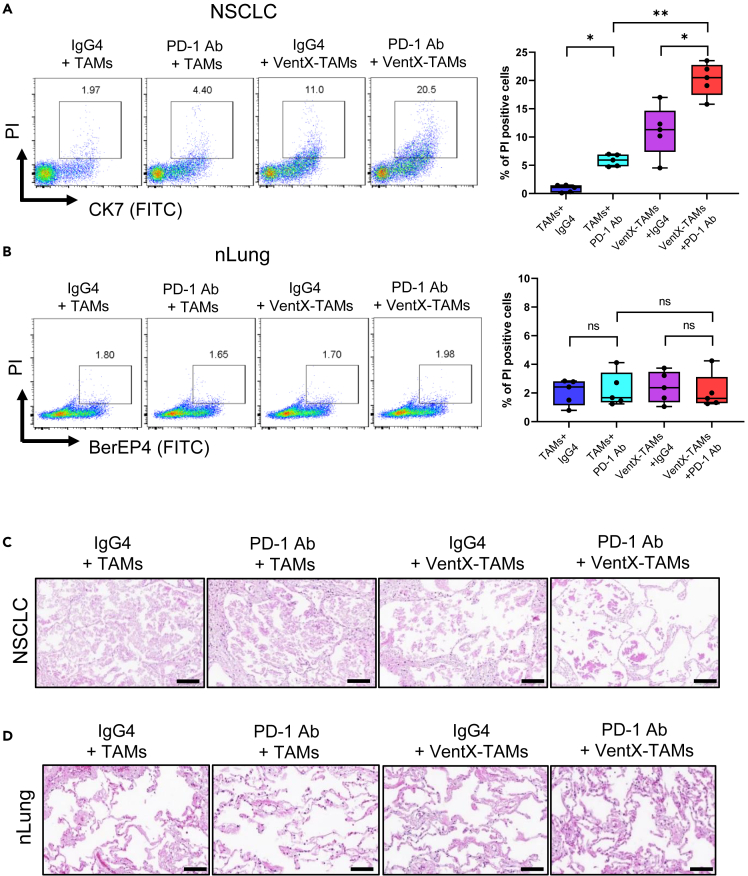
VentX-TAMs promotes tumoricidal effects of PD-1 antibodies in NSCLC-TME (A and B) *En bloc* NSCLS (A) or control nLung tissues (B) were treated with 1 μg/mL Pembrolizumab or control human IgG4 antibody and co-cultured with autologous TAMs transfected with GFP-VentX or control GFP for 5 days. Single cell suspensions were then obtained by mechanical disruption. The tumor cells were stained with CK7 antibodies and non-tumor epithelial cells were stained with EP4 antibodies. The cells were then labeled with PI and percentage of PI positive cells were determined by FACS analysis. Data represent means ± SD of 4 independent experiments. ∗ indicates p < 0.05, ∗∗ indicates p < 0.01 by one-way ANOVA analysis. The “ns” stands for no statistical significant difference. (C and D) Representative image of H&E staining of the tumor (C) or nLung tissues (D) after treatment. Scale bars: 100 μM.

**Figure 5:**
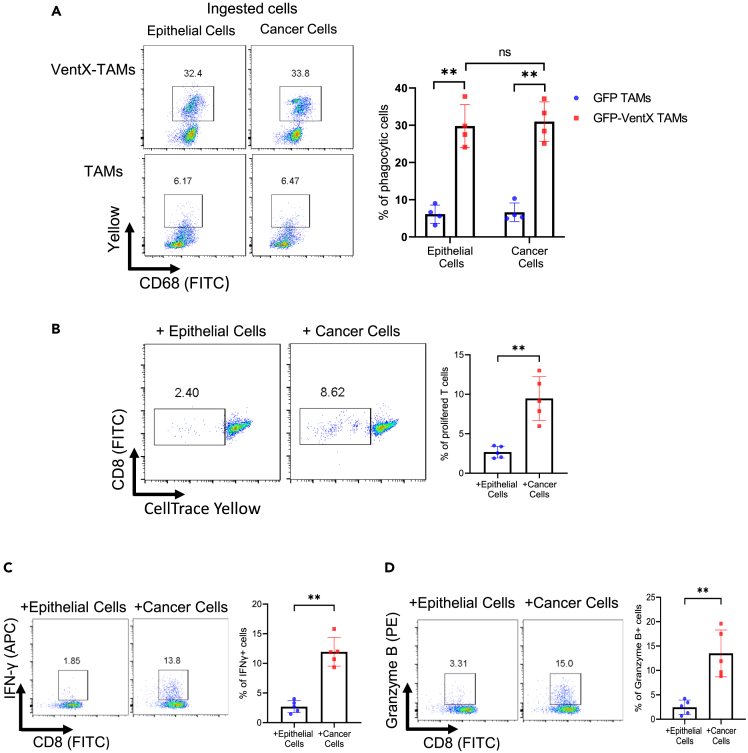
VentX-TAMs promotes CD8 T cell activation in cancer specific manner (A) VentX promotes TAM phagocytosis of cancer and normal epithelial cells. NSCLC-TAMs were isolated and transfected with plasmids encoding GFP or GP-VentX. The transfected TAMs were then incubated with 1 μM CellTrack Yellow-labeled cancer or normal epithelial cells at 1:1 ratio for 24 h. The rate of phagocytosis was then determined by flow cytometry. Data represent mean ± SD of 4 independent experiments. The “ns” stands for no statistical significant difference, ∗∗ indicates p < 0.01 by one-way ANOVA analysis. (B) Cancer specific stimulation of CD8 T cells proliferation by VentX-TAMs. VentX-TAMs were mixed with cancer or normal epithelial cells for 24 h and then co-cultured with CellTrack Yellow-labeled autologous CD8^+^ TIL at a ratio of 1:10 (M:T) for 5 days. The effects of the incubation on CD8 T cell proliferation were determined by FACS analysis. A representative figure was shown. (C and D) Cancer specific activation of CD8 T cells by VentX-TAMs. VentX-TAMs were mixed with cancer or normal epithelial cells for 24 h and then co-cultured with autologous CD8^+^ TIL at a ratio of 1:10 (M:T) for 5 days. The effects of the incubation on CD8 T cell activation were determined by FACS using APC-conjugated anti-IFNγ (C) or PE-conjugated anti-Granzyme B antibodies (D). Data represent the mean ± SD from 5 independent experiments, ∗∗ indicates p < 0.01 by paired Student’s *t* test.

Clinically, PD-1 antibody treatment elicits an overall response in about 20% of patients with NSCLC. Our findings that PD-1 antibody activates CD8 T cells in the *en bloc* NSCLC culture made us wonder whether *en bloc* NSCLC could be employed to evaluate the tumoricidal efficacy of PD-1 antibody against NSCLC *ex vivo*. To test this possibility, PD-1 antibody was applied to *en bloc* NSCLC and nLung tissue cultures for 5 days and the viability of the NSCLC cancer and normal lung epithelial cells was determined by PI staining and FACS analysis. We found that consistent with clinical outcomes, the application of anti-PD-1 antibodies to *ex vivo en bloc* NSCLC culture led to a modest increase of PI-staining of tumor cells but not the normal epithelial cells of nLung tissues (Figures S4A and S4B). As TME has been implicated in ICI resistance, we asked whether reversal of immune suppression in the NSCLC-TME would increase the efficacy of PD-1 antibodies. *En block* NSCLC or control nLung tissues were treated with PD-1 or control antibodies and co-cultured with autologous TAMs transfected with VentX or control GFP. The effects of co-culture were determined by FACS analysis of cancer or normal epithelial cell viability. We found that VentX-TAMs promote the cytocidal effect of PD-1 antibody against NSCLC in TME for about 4-fold; however, there was no significant increase in the death of normal epithelial cells (Figures 4A–4D). As VentX has been shown to modulate phagocytosis,^22^ to elucidate the mechanisms of VentX-TAM in promoting efficacy of PD-1 antibody against NSCLC, we hypothesized that VentX promotes NSCLC-TAM phagocytosis of NSCLC tumor cells, which in turn, promotes CD8 T cell activation in a cancer-specific manner. To this end, TAMs were transfected with GFP or GFP-VentX and then incubated with CFSE-labeled purified NSCLC cancer cells or normal epithelial cells (Figure S5) for 24 h. The effects of VentX on TAMs phagocytosis were determined by FACS analysis. As shown in Figure 5A, VentX promotes phagocytosis of both cancer and normal epithelial cells. To determine whether VentX could promote the TAM activation of CD8 T cells through cross-priming, after the phagocytosis step, we incubated VentX-TAMs with autologous CD8 T cells. Consistent with a cross-priming function of VentX-TAMs, we found that phagocytosis of cancer cells but not normal epithelial cells by VentX-TAMs led to about 4-fold enhancement of CD8 T cell proliferation and activation (Figures 5B–5D). Our findings suggest potential applications of the *en bloc* tumor and TAM co-culture models as a tumor immune microenvironment enabling model system (TIME-EMS) to explore the mechanisms and function of ICI in the context of the TME.

### VentX-regulated-tumor associated macrophages promote efficacy of PD-1 antibody against non-small cell lung cancer *in vivo*

The NSG models of patient-derived xenograft (NSG-PDX) are becoming important tools for cancer drug development.^37^ Compared with syngeneic mouse models, NSG-PDX models of primary human tumors have the advantage of bearing an immune relevant human TME and have been used successfully to evaluate the effects of modified TAMs on tumorigenesis of CRC and PDA^22^^,^^23^^,^^33^^,^^38^ (for Review^37^). Whether the NSG-PDX models of primary NSCLC can be used to evaluate the function of PD-1 antibody against NSCLC *in vivo* remains underexplored. To test that possibility, individual NSG-PDX models of primary NSCLC were generated by engrafting small pieces of primary NSCLC tissues into the subcutaneous space on the dorsal lateral side of NSG mice. Tumor growth was observed for up to 6 weeks and then the experiments were terminated due to the size limit of control tumors following the ethical guideline (Figure S6). For the rationale of using the models to test PD-1 antibody function *in vivo*, we sought to determine the presence of human CD8 T cells. To this end, successfully engrafted tumors were excised and the presence of primary human CD8 T cells within the implant were determined by FACS analysis. We found that the engrafted NSCLC tumors contain significant numbers of human CD8 T cells (Figure S6). Consistent with the presence of functional CD8 T cells in the implanted tumors, we found that the infusion of PD-1 antibody caused modest inhibition of tumorigenesis in these individual NSG-PDX models of primary NSCLC (Figure S6).

**Figure 6:**
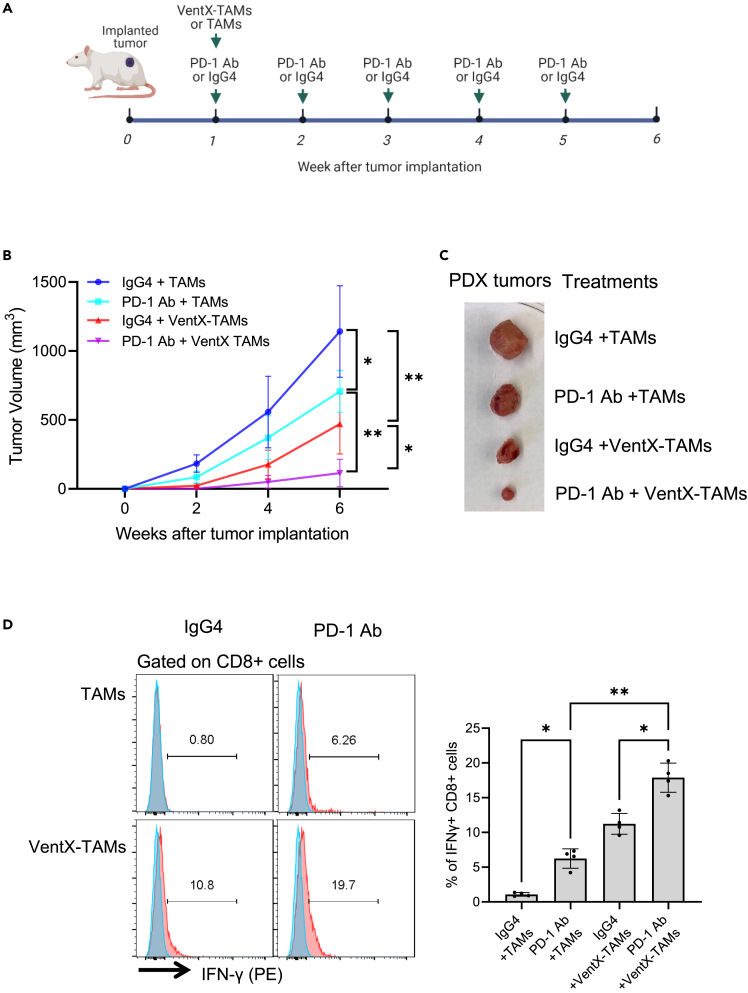
VentX-TAMs promotes efficacy of PD-1 antibody against NSCLC tumorigenesis in NSG-PDX models of individual primary NSCLC (A) Schematic of VentX-TAMs and PD-1 antibody or controls treatment of NSG-PDX models of primary NSCLC. (B) Growth curve of primary NSCLC in NSG-PDX mice treated with VentX-TAMs and PD-1 antibodies or controls as indicated. Data represent means ± SD of four independent experiments (n = 4), Statistical analysis were performed using two-way ANOVA with multiple comparison, ∗p < 0.05 ∗∗p < 0.01. (C) Representative images of excised tumors after 6 weeks of treatment as indicated. (D) The activities of CD8^+^ CTLs in PDX tumors. The PDX tumors were excised two-week post treatment. CD8^+^ CTLs were isolated by mechanical disruption and the CTL activities were determined by FACS analysis of IFNγ expression levels. Data shown are means ± SD of 4 independent experiments, and one-way ANOVA analysis was performed. ∗ indicates p < 0.05 and ∗∗ indicates p < 0.01.

**Figure 7:**
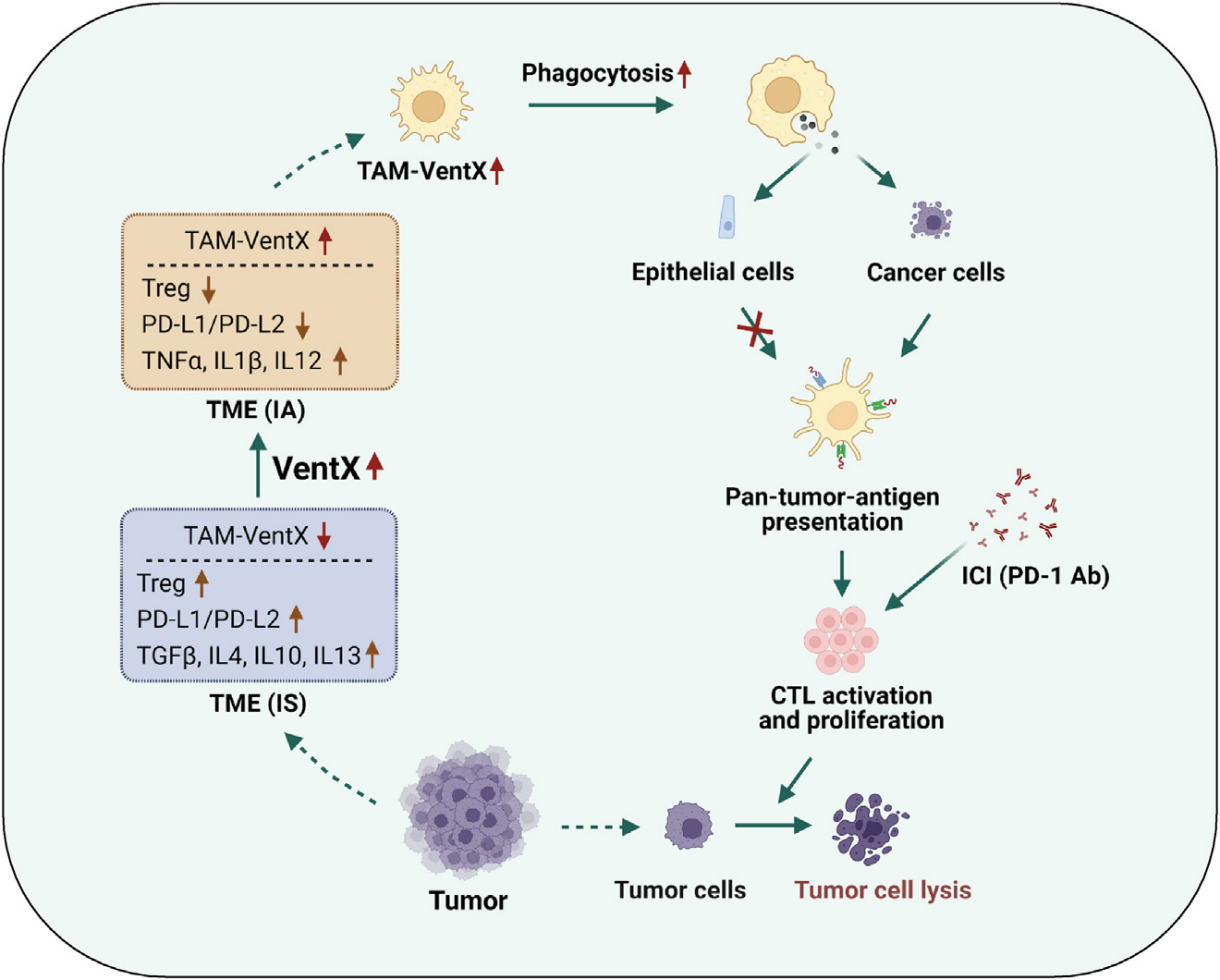
Schematic representation of VentX’s switch-like transformation of the tumor immune-landscape and overarching effects on CTL activation and ICI tumoricidal effects Elevated expression of VentX in TAMs polarizes TAMs from a pro-tumor phenotype to an anti-tumor phenotype and converts TME from immune suppressive (IS) to immune activation (IA) state. VentX promotes TAM phagocytosis, which in turn leads to CTL activation in a tumor specific manner and promote tumoricidal effects of ICIs.

To further evaluate the effects of VentX-TAMs on the PD-1 treatment of NSCLC *in vivo*, NSG-PDX models of primary human NSCLC were treated by the tail-vein injection of TAMs transfected with VentX or control GFP combined with the weekly injection of PD-1 or control antibody for 5 weeks. The effects were observed for 6 weeks (Figures 6A and S7). As shown in Figures 6B and 6C, VentX-TAMs promote the efficacy of PD-1 antibody against tumorigenesis of NSCLC for about 4-fold. Our previous studies showed that after tail-vein injection, the VentX-TAMs accumulate in tumors.^22^^,^^23^ To determine the mechanism underlying VentX-TAM enhancement of PD-1 antibody effects, we excised the implanted tumors two weeks post treatment and analyzed their cellular composition. We found that the infusion of VentX-TAMs promoted the PD-1 antibody activation of endogenous CD8 T cells within the implanted tumors for about 4-fold (Figure 6D). Together with the findings of the TIME-EMS studies, our data suggest that VentX-TAMs promote the efficacy of PD-1 antibody against NSCLC by reversing immune suppression in the TME (Figure 7).

## Discussion

Tumor antigen recognition and CTL activation are at the forefront of cancer immunotherapy.^39^^,^^40^ While the stimulation of CD8 T cells by ICIs have achieved unprecedented success in cancer immunotherapy, ICI efficacy against solid tumors remains limited and ICI-induced immune-related adverse events (irAE) can be severe.^34^ Recent development of mechanism-driven predictive biomarkers have provided encouraging results for optimizing individual response to ICI treatment,^41^ however, the overall response rate of solid tumors to ICI remains low. Inadequate antigen presentation and immune suppression of CD8 T cells within the tumor microenvironment (TME) have been proposed as two possible reasons for ICI treatment failure.^9^ To address these issues, extensive efforts have been focused on macrophages, the central executor of immunity.^42^^,^^43^ Tumor associated macrophages (TAMs) were found to be a major component of TME and have been proposed as and an ideal target of intervention to improve ICI efficacy.^44^^,^^45^ Nonetheless, without a practical platform to dissect the dynamic interaction between ICIs and immune cells within TME, the potential of TAM-targeting strategies has been limited by both the knowledge gap in macrophage regulatory mechanisms as well as the monetary and time demanding nature of clinical trials.^2^^,^^46^

Formation of dorsoventral asymmetry within the symmetrical structure of an embryo is the consequence of coordinated cell proliferation and differentiation during early embryogenesis. It provides the foundation for ontogenesis of tissues and organs and has broad implications in clinical medicine. Regarding the mechanisms of signal integration that lead to the formation of the dorsoventral axis during early vertebrate embryogenesis, our previous work on *Xenopus* development showed that the dorsal cell fate determination factor beta-catenin and the ventral cell fate determination factor Xom converge onto the common high motility group (HMG) box containing transcription factor LEF/TCFs to exert their integrated antagonizing effects on cell fate.^18^^,^^47^ Over the past two decades, in addition to our interests in the role and mechanism of ubiquitin-mediated proteolysis in governing the dynamic balance between beta-catenin and Xom in the establishment of dorsoventral axis formation,^16^^,^^48^ we endeavored to translate the significance of developmental findings into potential clinical impact. We searched for and identified the homeobox protein VentX, a human Xom homologue, as a key controller of the proliferation and differentiation of human hematopoietic cells. We found that like Xom, VentX binds to LEF/TCFs and antagonizes the oncogenic Wnt signaling during leukemogenesis.^19^ Further physiological and mechanistic investigation showed that VentX controls the proliferation and differentiation of human hematopoietic cells by modulating cell cycle machinery and signaling pathways, such as the Cyclin D1, c-MYC, p53, p21 and p16^ink4a^^19^^,^^21^^,^.^49^ In addition to its role in blood ontogenesis and leukemogenesis, our studies showed that VentX also plays important role in governing monocyte differentiation through modulating critical signaling involved in the process, such as the M-CSF signaling.^20^ Moreover, we found that VentX controls macrophage plasticity and promotes the expression of pro-inflammatory M1 cytokines and cell surface markers but it inhibits the expression of pro-regulatory M2 cytokines and cells surface markers.^20^ More recently, VentX was found to play important role in governing the phagocytic function of macrophages by controlling the SIP1, SIP2, FAK kinase and the TLR4 and TLR9, but not the SIRPα.^22^ The role of VentX in immunopathogenesis of cancer was first indicated by our findings that VentX expression is down-regulated in TAMs.^23^ Consistent with the notion that TAMs play an important role in governing immunological state of TME, we found that down-regulated VentX expression in TAMs underlies the immune suppression of TME by controlling the proliferation and differentiation of TILs.^22^^,^^23^ As immune suppressive TME has been implicated in cancer chemo-resistance, we developed an *en bloc* tumor and TAMs co-culture model as a tumor immune microenvironment enable model system (TIME-EMS) to explore the effects of VentX-modulated-TIME in promoting chemosensitivity.^24^ In the current study, we tested the feasibility of employing the TIME-EMS to explore the tumoricidal function of PD-1 antibody against NSCLC in the context of NSCLC-TME. The authenticity of the model in reflecting the physiological function of TME in ICI treatment was indicated by our finding that the activation of CD8 T cells in NSCLC-TME was dosage-dependent and that, different from cell-based *in vitro* assays, no antigens or cytokines are needed for the PD-1 antibody activation of CD8 T cells in the TME.^32^^,^^34^^,^^50^ Using the TIME-EMS models, we demonstrated that reprogramming TME by VentX-modulated-TAMs (VentX-TAMs) enhance the reinvigoration of CD8 T cells and the cytocidal effects of PD-1 antibody against NSCLC approximately 4-fold, but not the cytocidal effects on normal lung epithelial cells (Figure 4). Our previous studies showed that VentX drives the phagocytotic function of TAMs.^22^ Mechanistically, consistent with the idea that phagocytosis plays an important role in cancer immune surveillance,^51^ the down-regulated VentX expression in TAMs suggested that the lack of an internal drive of TAM for phagocytosis may play an important role in the immune evasion of cancer cells. As such, restoring of VentX expression in TAMs may lead to the recovery of its phagocytic function and the return of its immune surveillance capability. This approach offers an opportunity for the TAMs to present pan-tumor-antigens (PTAs) to CTLs, and consequently activates CTLs in a tumor specific manner (Figure 7). CTL activation by PTAs diminishes the possibility of immune escape frequently encountered during immune stimulation by individual tumor antigens and reduces the risk of irAE associated with the non-discriminating activation of immune system by ICIs. Using the TIME-EMS as a testing model, our data provided the long-sought-after evidence of TAM cross-priming and activating TILs and the results are consistent with the idea of pre-existing pools of tumor-specific CD8 T cells in the TME.^3^^,^^46^^,^^52^

The VentX is a unique human homeobox protein that lack a murine homologue.^53^ As such, to explore the potential therapeutic function of VentX-TAMs *in vivo*, we developed the NSG-PDX models of primary human cancers as an unique pre-clinical testing model.^23^^,^^37^ In Comparison with the commercial available NSG-PDX models of human cancers, which are generated by the serial passaging of implanted human tumors in NSG mice, the NSG-PDX models of primary human cancers have the advantage of carrying a human cancer with relatively preserved human TME. In the current study, using the NSG-PDX models of primary human NSCLC, we demonstrated that VentX-TAMs promote the efficacy of ICI against NSCLC *in vivo* approximately 4-fold. The results suggested the role of VentX as a target of intervention and the potentials of the genetic or chemical modification of VentX expression in cancer immunotherapy. In the current study, we used the NSCLC as a model to illustrate the function of VentX in modulating TIME to promote efficacy of ICIs. In addition to NSCLC, our preliminary studies suggested that such a mechanism may also be applicable for immunotherapy of other solid tumors, such as colorectal cancer. Taken together, our work demonstrates that detailed studies of regulatory processes in embryogenesis can serve as a powerful source of potential mechanisms for regulating dysgenesis, a key hallmark of cancer. The unexpected repurposing of developmental pathways for regulation in adult tissues is well established in these studies and in many others such as the Wnt pathway.

Interestingly, opposite to the down-regulated expression of VentX in TAMs and its implications in the immunopathogenesis and treatment of cancer, elevated VentX expression in mononuclear phagocytes has been implicated in pathogenesis and treatment of autoimmune inflammatory disorders, such as RA/SLE and IBD.^20^^,^^36^ As such, consistent with the role of macrophage as a central executor of immunity and the function of VentX in governing macrophage polarity, our studies suggested that regulated expression of VentX in macrophage plays a critical role in modulating immune polarity in pathogenesis and treatment of autoimmune inflammatory disorders and cancer.

### Limitations of the study

Our current study suggests the role of VentX-regulated-TAMs in promoting the efficacy of ICIs against solid tumors through mechanisms of tumor-specific activation of cytotoxic lymphocytes. However, the mechanisms of VentX enhancement of TAM tumor antigen presentation and tumor-specific action of CTLs remain to be further determined and the potential benefits of these findings for patients with cancer remain to be tested by rigorous clinical trials.

## STAR★Methods

### Key resources table

**Table:** 

REAGENT or RESOURCE	SOURCE	IDENTIFIER
**Antibodies**		

Mouse monoclonal anti-CD4 (OKT-4), FITC	Thermo Fisher Scientific	Cat# 11-0048-42, RRID:AB_1633390
Mouse monoclonal anti-CD4 (OKT-4), PE	Thermo Fisher Scientific	Cat# 12-0048-42, RRID:AB_2016675
Mouse monoclonal anti-CD8 (SK1), FITC	BioLegend	Cat# 344704, RRID:AB_1877178
Mouse monoclonal anti-CD8 (SK1), PE	BioLegend	Cat# 344706, RRID:AB_1953244
Mouse monoclonal anti-CD11B (ICRF44), FITC	Thermo Fisher Scientific	Cat# 11-0118-42, RRID:AB_1582243
Mouse monoclonal anti-CD14 (61D3), FITC	Thermo Fisher Scientific	Cat# 11-0149-42, RRID:AB_10597597
Mouse monoclonal anti-CD25 (BC96), PE	Thermo Fisher Scientific	Cat# 12-0259-42, RRID:AB_1659682
Mouse monoclonal anti-CD40 (5C3), PE	Thermo Fisher Scientific	Cat# 12-0409-42, RRID:AB_1963582
Mouse monoclonal anti-CD68 (Y1/82A), FITC	Thermo Fisher Scientific	Cat# 11-0689-42, RRID:AB_11149303
Mouse monoclonal anti-CD80 (2D10.4), PE	Thermo Fisher Scientific	Cat# 12-0809-42, RRID:AB_1311209
Mouse monoclonal anti-CD152 (CTLA-4) (14D3), FITC	Thermo Fisher Scientific	Cat# 11-1529-42, RRID:AB_2637119
Mouse monoclonal anti-CD163 (GHI/61), PE	Thermo Fisher Scientific	Cat# 12-1639-42, RRID:AB_1963570
Mouse monoclonal anti-CD206 (MMR) (19.2), PE	Thermo Fisher Scientific	Cat# 12-2069-42, RRID:AB_10804655
Mouse monoclonal anti-CD273 (PD-L2) (MIH18), PE	Thermo Fisher Scientific	Cat# 12-5888-42, RRID:AB_10853342
Mouse monoclonal anti-CD274 (PD-L1) (MIH1), PE	Thermo Fisher Scientific	Cat# 12-5983-42, RRID:AB_11042286
Mouse monoclonal anti-CD279 (PD-1) (J105), PE	Thermo Fisher Scientific	Cat# 12-2799-41, RRID:AB_10597751
Mouse monoclonal anti-FOXP3 (236A/E7), APC	Thermo Fisher Scientific	Cat# 17-4777-42, RRID:AB_10804651
Mouse monoclonal anti-IFNγ (4S.B3), FITC	Thermo Fisher Scientific	Cat# 11-7319-41, RRID:AB_11043263
Mouse monoclonal anti-Granzyme B (GB11), PE	Thermo Fisher Scientific	Cat# 12-8899-41, RRID:AB_1659718
Mouse monoclonal anti-IL17AF (20LJS09), PE	Thermo Fisher Scientific	Cat# 12-9179-42, RRID:AB_10852705
Mouse monoclonal anti-Cytokeratin 7 (5D12)	Thermo Fisher Scientific	Cat# MA5-15604, RRID:AB_10981428
Mouse monoclonal anti-Epithelial Antigen (Per-EP4)	Agilend	Cat# M0804, RRID:AB_2335685
Pembrolizumab (humanized anti-PD-1 antibody, IgG4)	Selleckchem	Cat# A2005,
Goat anti-mouse IgG (H + L) secondary antibody, FITC	Thermo Fisher Scientific	Cat# 62–6511, RRID:AB_2533946

**Biological samples**		

Human lung cancer and their adjacent tissues	Brigham and Women’s Hospital	N/A
The blood samples	Brigham and Women’s Hospital	N/A

**Chemicals, peptides, and recombinant proteins**		

Ficoll-Paque plus	Cell Signaling Tech.	Cat# 5872
Collagenase D	Roche	Cat# 11088858001
Trizol reagent	Life technologies	Cat# 15596026
Propidium Iodide staining solution	Thermo Fisher Scientific	Cat# 00-6990-50
Isothesia (Isoflurane, USP)	Henry Schein	Cat# 11695-6776-1

**Critical commercial assays**		

CellTrace yellow cell proliferation kit	Thermo Fisher Scientific	Cat# C34567
Human macrophage nucleofector kit	Lanzo	Cat# F12796
Griess reagent Kit	Thermo Fisher Scientific	Cat# G7921
EasySep human monocyte/macrophage kit	Stemcell Technologies	Cat# 19359
EasySep human CD8 T cell kit	Stemcell Technologies	Cat# 17953
Human IL-1 beta ELISA kit	Thermo Fisher Scientific	Cat# 88-7261
Human IL-12 p70 ELISA kit	Thermo Fisher Scientific	Cat# 88-7126
Human TNF alpha ELISA kit	Thermo Fisher Scientific	Cat# 88-7346
Fixation/Permeabilization buffer set	Thermo Fisher Scientific	Cat# 88-8823-88
Superscript III reverse transcript kit	Thermo Fisher Scientific	Cat# 18080-051
SimpleChip Plus Enzymatic Chromatin IP kit	Cell Signaling Technology	Cat# 9004

**Experimental models: Organisms/strains**		

NSG mouse: NOD.Cg-*Prkdc<scid> Il2rg<tm1Wjl>/SzJ*	Jackson Laboratory	Cat# 005557

**Oligonucleotides**		

The primers for RT-PCR are listed in Table S1	Thermo Fisher Scientific	N/A
Morpholino: VentX-MO; (TACTCAACCCTGACATAGAGGGTAA)	Gene Tools	N/A

**Recombinant DNA**		

pCS2-GFP-VentX plasmid	In our lab	N/A
pCS2-GFP plasmid	Addgene	Cat# 15681

**Software and algorithms**		

GraphPad prism v9.5.1	Dotmatics	N/A
ImageJ	NIH Image	https://imagej.nih.gov/ij/
FlowJo v10.7	Flowjo LLC	N/A
CaseViewer	3DHISTECH Ltd.	N/A

### Resource availability

#### Lead contact

Further information and requests for resources, reagents should be directed to and will be fulfilled by the lead contact, Zhenglun Zhu (zzhu@bwh.harvard.edu).

#### Materials availability

This study did not generate new unique reagents.

#### Data and code availability

• This study does not report the original code.
• Data reported in this paper will be shared by the lead contact upon request.
• Any additional information required to reanalyze the data reported in this paper is available from the lead contact upon request.

### Experimental model and subject details

#### NSG-PDX model of primary human NSCLC

Animal models of primary human NSCLC were developed in the laboratory. Both male and female mice were used. All animal experiments were approved by the Institutional Animal Care and Use Committee at Brigham and Women’s Hospital. Briefly, 8-week-old NOD.Cg-Prkdcscid Il2rgtm1Wjl/SzJ mice (commonly known as NOD scid gamma, or NSG mice) were purchased from Jackson Laboratory and maintained under specific pathogen-free conditions. Tumors were cut into around 0.5 cm pieces and then surgically implanted into subcutaneous spaces in the flanks of NSG mice. One week after implantation, 0.25 × 10^6^ TAMs transfected with GFP-VentX or control GFP were injected into the mice through the tail vein. Tumor growth was monitored twice weekly and measured using a caliper for 6 weeks. Tumor volumes were calculated according to the formula ½ (length × width^2^).

#### Study approval

All animal experiments were approved by the Institutional Animal Care and Use Committee at Brigham and Women’s Hospital; Animal Approval Number 2016N000353.

#### Collection of human tissue samples

A total of 24 patients with NSCLC, who were scheduled for surgical resection at Brigham and Women’s Hospital, were consented to have a portion of resected tissues and blood collected for research purposes. All patients signed an informed consent document that was approved by the Institutional Review Board of Brigham and Women’s Hospital. The characteristics of the lung cancer patients whose specimens were used for this study were listed in the table below. Around 5-10gram tissues were collected from tumor mass, or non-involved lung tissues. Tumor samples and control tissues were verified by board certified pathologists at the institution.

**Table:** Characteristics of patients with NSCLC and specimens

Characteristics	Number (n = 24)	(%)
**Age, y**		

40–60	9	37.5
60–80	15	62.5

**Sex**		

Male	14	58.3
Female	10	41.7

**Pathology type**		

Adeno	18	75.0
Squamous	6	25.0

**Stage**		

I	9	37.5
II	7	29.1
III	6	25.0
IV	2	8.33

#### Study approval

The study using discarded human material was approved by the Institutional Review Board of Brigham and Women’s Hospital, Boston, MA. Tumor samples and control tissues were verified by board-certified pathologists at the institution. The IRB study Number is 2006P1354.

### Method details

#### Preparation of lymphocytes and macrophages from tumor tissues

Lymphocytes were isolated essentially as described.^20^^,^^23^^,^^54^ Briefly, dissected fresh tumor and lung tissues were rinsed in 10-cm Petri dish with Ca^2+^-free and Mg^2+^-free hank’s balanced salt solution (HBSS) (life technologies) containing 2% fetal bovine serum (FBS) and 2 mM Dithiothreitol (DTT) (Sigma-Aldrich). The lung and tumor tissues were then cut into around 0.1 cm pieces by a razor blade and incubated in 5 mL HBSS containing 5 mM EDTA (Sigma-Aldrich) at 37°C for 1 h. The tissues were then passed through a gray-mesh (100 micron). The flow-through containing lymphocytes and epithelial cells were then analysis by a flow cytometer.

To isolate the macrophages, tumor and lung tissues were rinsed with HBSS, cut into around 0.1 cm pieces by a razor blade and then incubated in HBSS (with Ca^2+^ and Mg^2+^), containing 2% FBS, 1.5 mg/mL Collagenase D (Roche), 0.1 mg/mL Dnase I at 37°C for 1 h. The digested tissues were then passed through a gray-mesh (70 micron) filter. The flow-throughs were collected, washed, and resuspended in RPMI 1640 medium. Normal tissue macrophages and TAMs were further purified using EasySep Human Monocyte/Macrophage Enrichment kit without CD16 depletion (StemCell Technologies, Cat# 19085) according to the manufacturer’s instructions. The isolation process does not lead to activation of macrophages and the purity of isolated macrophages was above 95%.^20^^,^^54^^,^^55^ More than 98% of cells isolated by the techniques were viable by propidium iodide (PI) staining tests.

#### FACS analysis

Phenotypic analysis of macrophages and lymphocytes was performed using flow cytometry after immunolabeling of cells with fluorescence dye–conjugated antibodies. The antibodies used were listed in Table S1. Extracellular staining was performed at 4°C for 30 min and then fixed with 2% paraformaldehyde. For intracellular staining, the cells were fixed and permeabilized using fixation/permeabilization solution (Fisher Scientific) following the protocol provided by the manufacturer and then subjected to antibody staining. Isotope control labeling was performed in parallel. Antibodies were diluted as recommended by the supplier. For experiments involve propidine iodide (PI) staining, after the staining step, the cells were washed and resuspended in 200 μl of FACS staining solution supplemented with 5 μl of PI staining solution (eBioscience/Fisher Scientific) for 15 min before subjected to FACS analysis. Labeled cells were acquired using the BD LSRFortessa at the Flow Cytometry Core of the Dana Farber Cancer Institute with the FACS Diva software (BD Biosciences) and analyzed using the FlowJo 10.1 software (Treestar). Typically, 20,000 cells were analyzed per sample according to the standard FACS analysis procedure. Compensation was performed with two or more fluorescence of antibody staining and the instrument was calibrated daily using CS&T beads. Gating was performed on life single cells. Results are expressed as the percentage of positive cells.

#### Quantitative RT-PCR

Total RNA was isolated by the TRIzol reagent (Life Technologies) and RNA amounts were measured by NanoDrop 2000 (Thermo Scientific). Equal amount of RNA was used for first-strand cDNA synthesis with SuperScript III First-Strand Synthesis System (Life Technologies) according to the manufacturer’s protocol. The AccuPrime Taq DNA polymerase system (Life Technologies) was used to amplify VentX cDNA with conventional PCR. Quantitative measurements of VentX and other cDNA were carried out with SYBR Green, using a Mastercycler ep Gradient S (Eppendorf). GAPDH was used as a house keeping gene to normalize mRNA expression. The primers used were listed in Table S2. Relative expression profiles of mRNAs were calculated using the comparative Ct method (DDCT method).

#### Cytokine measurement

Levels of IL-1β, IL-2, IFN-γ and TNF-α were quantified using ELISA kits obtained from eBiosciences. Analyses were conducted according to the manufacturer’s instructions. Triplicate wells were plated for each condition.

#### Transfection assays

Plasmids encoding GFP-VentX were generated by inserting the VentX gene at the multicloning site in frame following the GFP gene, using the pCS2-eGFP vector (Addgene).^19^ Ectopic expression of GFP-VentX or control GFP in TAMs were made by transient transfection of plasmids encoding GFP-VentX or the control GFP, using the Human Macrophage Nucleofector Kit (Catalog #: VVPA-1008, Lonza, Walkersville, MD). Briefly, 2×10^6^ cells were re-suspended into 100 μL nucleofector solution with 5 μg of plasmid DNA for 20 min on ice. Transfections were performed in Nculeofector 2b Device (Lonza). After transfection, cells were placed on ice immediately for 1 min and then cultured in pre-warmed RPMI 1640 complete medium, containing 10% FBS and 1% antibiotic-antimycotic solution (Gibco, Cat# 15240062) for 24–48 h before transfected cells were used for experiments.

#### *En bloc* tissue culture and treatment

Tumor tissue were washed with 1× PBS buffer plus antibiotics and then cut into 0.5 cm pieces. Tissues were cultured in 2 mL of RPMI 1640 medium, supplemented with 5% FBS (Sigma) and 1% antibiotic-antimycotic solution (Gibco) in 24-well plate (Corning). The cultures were incubated at a 37°C, 5% CO_2_ incubator. For functional evaluation of PD-1 antibody on *en bloc* tissue culture, Pembrolizumab (Humanized anti-PD-1 monoclonal antibody, Selleckchem) or human IgG4 isotype control at indicated concentration were added in the wells and the effects on immune cell activities and tumor cell survival were determined as described. For *en bloc* tissue and macrophage co-culture assay, 0.25 x 10^6^ GFP-VentX or control GFP transfected autologous macrophages of the same patient were added to the *en bloc* tissue culture wells. After gently shaking, the plate was incubated at 37°C, 5% CO_2_ for 3–5 days. The *en bloc* tissues were then subjected to single l cell isolation and analysis as described.

#### Isolation of tumor and normal epithelial cells

Tumor and normal lung tissues were cut into around 0.1 cm pieces by a razor blade and single cell suspensions generated by mechanical disruption followed by filtering of cell suspension through 70 μm nylon mesh. After washing with PBS, the cells were resuspended in RPMI only medium and were placed on the top of Ficoll solution in 15 mL Falcon tubes. The tubes were then centrifuged in Beckman Allegra 6R tabletop centrifuge at 2000 rpm for 30 min with low acceleration and deceleration. The cells were then collected from the bottom of Falcon tubes and the red blood cells were removed by RBC lysis buffer (Fisher Scientific). After washing with PBS, the tumor cells and normal epithelial cells were collected in RPMI complete medium. The isolated cells were further characterized by CK7 and EP4 antibody staining and FACS analysis.

#### Phagocytosis assays

NSCLC tumor cells or normal epithelial cells were labeled with 1μM of CellTrace Yellow using Cell Proliferation Kit (Fisher Scientific). The labeled cells were then mixed with 2×10^5^ autologous TAMs transfected with GFP or GFP-Ventx at 1:1 ratio in 12-well tissue culture plates (Coring) and incubated in RPMI complete medium, plus 10% FBS, 1% antibiotic-antimycotic solution (Gibco, Cat# 15240062). After 24 h incubation, the TAMs were washed and collected with a cell scraper and phagocytosis was analyzed by a flow cytometer.

#### T cell proliferation and activation assays

T cell proliferation and activation assays were performed essentially as described.^23^^,^^56^ For proliferation assays, the CD8^+^ TILs of the NSCLC patients were isolated by the Easysep human CD8 enrichment kit (StemCell Technologies, catalog 19053) following the manufacturer’s instructions and then labeled with 1 μM of CellTrace using Cell Proliferation Kit (Fisher Scientific). To prepare TAMs, 10^5^ of GFP-VentX or GFP transfected TAMs were mixed with same amount of tumor cells or normal epithelial cells from the same patient and cultured in 12-well plate with RPMI 1640 medium plus 10% FBS, 1% antibiotic-antimycotic solution (Gibco, Cat# 15240062) at 37°C, 5% CO_2_ for 24 h. The 1×10^6^ labeled CD8 TILs and the TAMs were then mixed at 10:1 ratio and then cultured at 37°C, 5% CO_2_ for 5 days. Cells were then stained with an anti–CD8-APC–conjugated antibody and analyzed by a flow cytometer. For activation assays, CD8^+^ TIL were mixed with treated TAMs at 10:1 ratio and then cultured at 37°C, 5% CO_2_ for3 days. The cells were then stained with an anti–CD8-APC–conjugated antibody and presence of intracellular INF-γ and Granzyme B were determined by FACS as described.

#### Individual NSG-PDX models of primary human NSCLC

Individual NSG-PDX models of primary human lung cancers were developed as described previously.^57^ All animal experiments were approved by the Institutional Animal Care and Use Committee at the Brigham and Women’s Hospital. Briefly, 8-week-old NOD.Cg-Prkdcscid Il2rgtm1Wjl/SzJ mice (commonly known as NOD scid gamma, or NSG mice) were purchased from the Jackson Laboratory and maintained under specific pathogen-free conditions. NSCLC tumors were cut into around 0.5 cm pieces and then surgically implanted into subcutaneous space on the dorsal side of NSG mice. One week after the implantation, the animals were treated with PD-1 antibody or VentX-TAMs or controls as indicated. For the PD-1 antibody treatment group, 150 μg of pembrolizumab (humanized anti-PD-1 antibody) or human IgG4 control were tail-vain injected once a week for up to 5 weeks as indicated; for the VentX-TAM treatment group, 0.25 × 10^6^ TAMs transfected with GFP-VentX or control GFP were injected through tail vain once as indicated. The tumor growth was monitored twice weekly and measured by a caliper for 6 weeks. Tumor volumes were calculated according to the formula ½ (length × width^2^).

#### Immunohistochemistry

Immunohistochemistry were performed following established protocol.^58^ Briefly, lung tumors or normal tissues were fixated in formalin (Fisher Scientific Company, Kalamazoo, MI) for at least 48 h. The tissues were then embedded in paraffin and sectioned. Haematoxylin/eosin (H&E) staining were performed at Specialized Histopathology Core at Dana-Farber/Harvard Cancer Center. The images of whole slides were scanned by Pannoramic MIDI II digital slide scanner and analyzed with Caseviewer and Quant center software (3DHistech).

### Quantification and statistical analysis

Student’s test or one-way ANOVA were used for statistical analysis in Prism version 9 (GraphPad, La Jolla, CA). Data are presented as mean ± standard deviation (SD). Tumor growth curves were analyzed by repeated measurement two-way ANOVA using Sidak’s multiple comparison test. The level of significance was indicated by the p value. In all figures, levels of statistical significance were indicated as: ∗p < 0.05, ∗∗p < 0.01.
