# Supplementary material for: VentX promotes tumor specific immunity and efficacy of immune checkpoint inhibitors
Source: iScience. 2023 Dec 14;27(1):108731. doi: 10.1016/j.isci.2023.108731 (PMC10829883; doi:10.1016/j.isci.2023.108731)
Supplement: Document S1. Figures S1–S7 and Tables S1–S3 [file mmc1.pdf]

iScience, Volume *27*

Supplemental information

VentX promotes tumor specific immunity and efficacy of immune checkpoint inhibitors

Yi Le, Hong Gao, Joanna Le, Jason L. Hornick, Ronald Bleday, Jon Wee, and Zhenglun Zhu

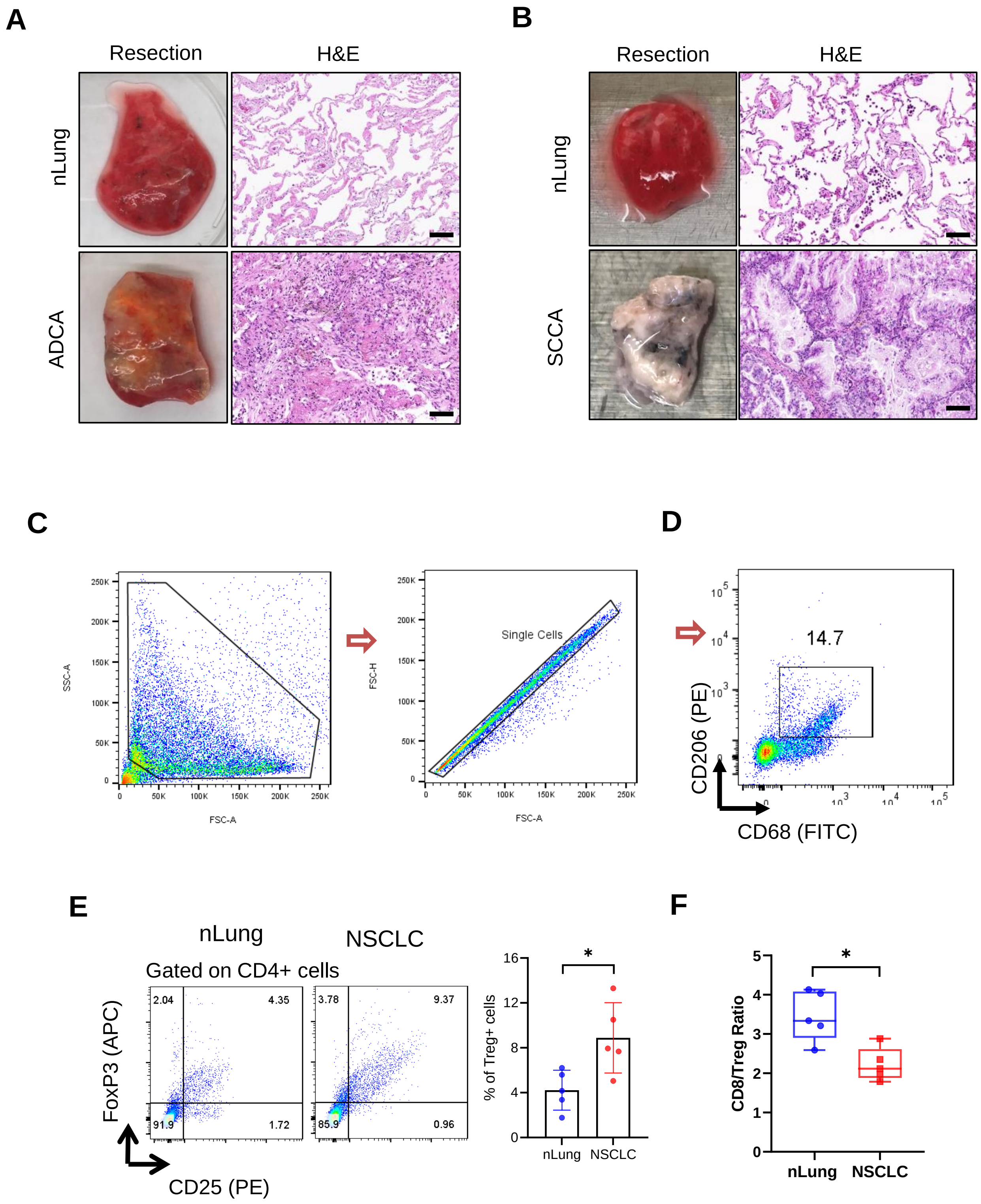
A
Resection
H&E
nLung
ADCA
B
Resection
H&E
nLung
SCCA
C
SSC-A
FSC-A
FSC-H
Single Cells
FSC-A
D
14.7
CD206 (PE)
CD68 (FITC)
E
nLung
NSCLC
Gated on CD4+ cells
2.04
4.35
91.9
1.72
3.78
9.37
85.9
0.96
FoxP3 (APC)
CD25 (PE)
% of Treg+ cells
*
nLung
NSCLC
F
CD8/Treg Ratio
*
nLung
NSCLC

Figure S1. Gating strategy and characterization of NSCLC tumor associated immune cells, related to Figure 1 . (A) *Gating strategy of FACS analysis. Mononuclear cells were gated on the basis of FCS-A versus SSC-A and then were sequentially gated to select single cells by FSC-A versus FSC-H. Subsequently, flow cytometry plots for the various markers were shown in biexponential format.* ***(B)*** *FACS analysis of NSCLC-TAMs, using CD68 and CD206 markers.* ***(C-F)*** *Characterization of Treg and CD8 cells isolated from nLung tissues and NSCLC.* ***(C)*** *CD8 T cells isolated from NSCLC and nLung tissues were stained with fluorescence conjugated PD-1 and CTLA4 antibodies (eBioscience/ Invitrogen) and subjected to FACS analysis.* ***(D)*** *Isolated CD8 T cells were stimulated with Dynabeads (Cells: Beads ratio is 5:1) for 48 hours at* $37^{0}C$*. Cells were then fixed, permeabilized and stained with anti-IFNγ and anti-Granzyme B PE-conjugated antibodies and subjected to analysis with a flow cytometer.* ***(E)*** *Percentage of Treg cells in NSCLC and nLung tissues. Isolated tissue mononuclear cells were stained with anti-CD4-FITC, CD25-PE, Foxp3-APC and subjected to FACS analysis. The percentage of Treg (CD4+, CD25+, Foxp3+) cells was shown. (**F**) CD8/Treg ratio in nLung and NSCLC tissues. Data represent means ± S.D. of five independent experiments, n=5, ** $p < 0.05$*, *** $p < 0.01$ *by paired Student's t test.*

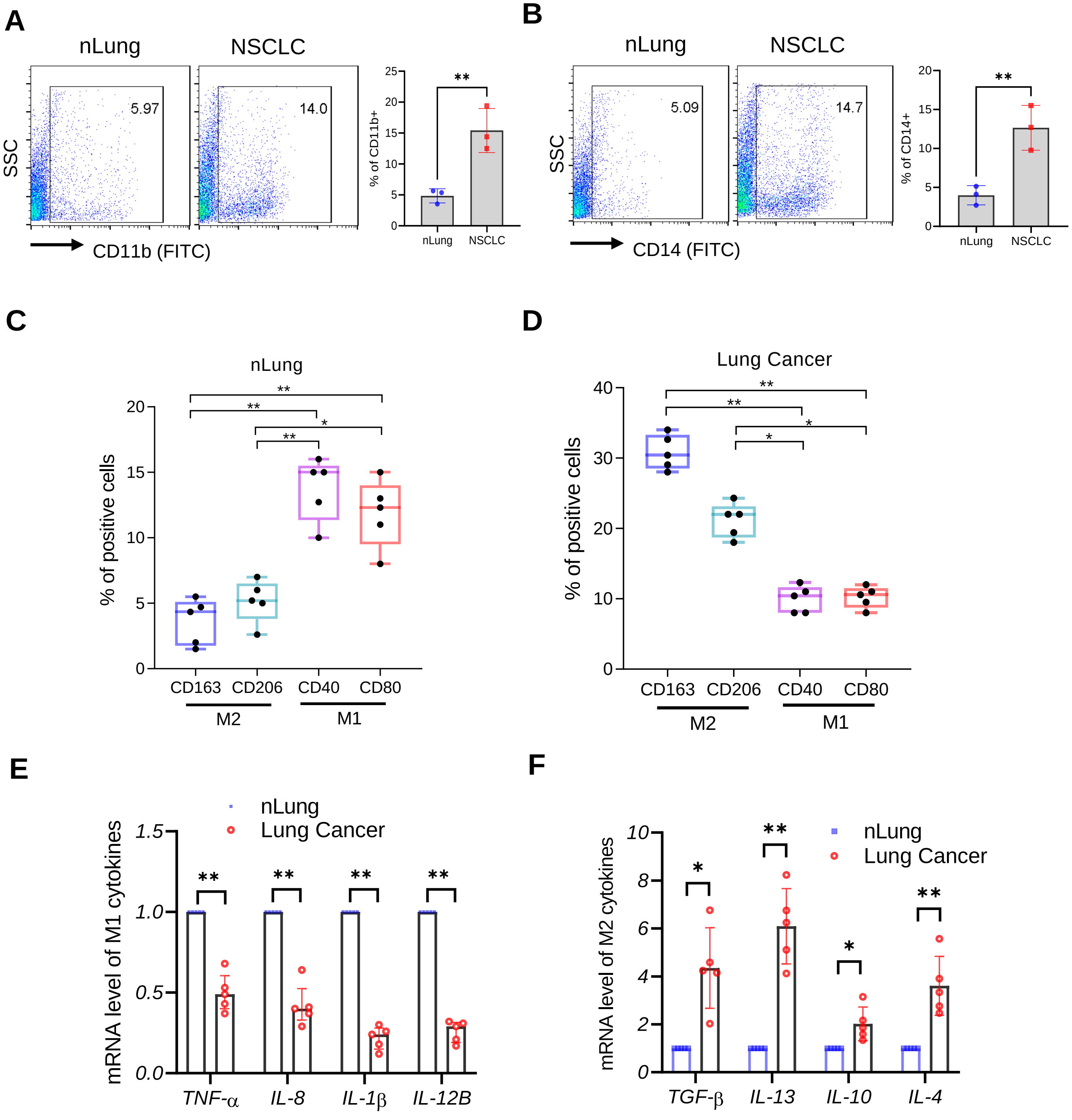
A
nLung
NSCLC
SSC
5.97
14.0
CD11b (FITC)
% of CD11b+
nLung
NSCLC
**
B
nLung
NSCLC
SSC
5.09
14.7
CD14 (FITC)
% of CD14+
nLung
NSCLC
**
C
nLung
% of positive cells
CD163
CD206
CD40
CD80
M2
M1
D
Lung Cancer
% of positive cells
CD163
CD206
CD40
CD80
M2
M1
E
nLung
Lung Cancer
mRNA level of M1 cytokines
TNF-α
IL-8
IL-1β
IL-12B
F
nLung
Lung Cancer
mRNA level of M2 cytokines
TGF-β
IL-13
IL-10
IL-4

Figure S2. Characterization of NSCLC-TAMs, related to Figure 1. (A, B) *Cell surface expression of macrophage markers CD11b* ***(A)*** *and CD14* ***(B)*** *from nLung and NSCLC. Results represent mean ± SD of three independent experiments.* ***(C, D)*** *Cell surface expression of M1- and M2- markers in macrophages isolated from nLung* ***(C)*** *and NSCLC* ***(D)*** *as determined by FACS analysis. Results represent mean ± SD of five independent experiments.* ***(E,F)*** *Quantification of M1* ***(E)*** *and M2* ***(F)*** *cytokine mRNA expression in macrophages isolated from nLung and NSCLC by qRT-PCR. The relative mRNA expression levels in control macrophages isolated from nLung were arbitrarily designated as 1. Results represent means ± SD of five independent experiments. * indicates* $p < 0.01$*, ** indicates* $p < 0.01$ *by paired Student's t test.*

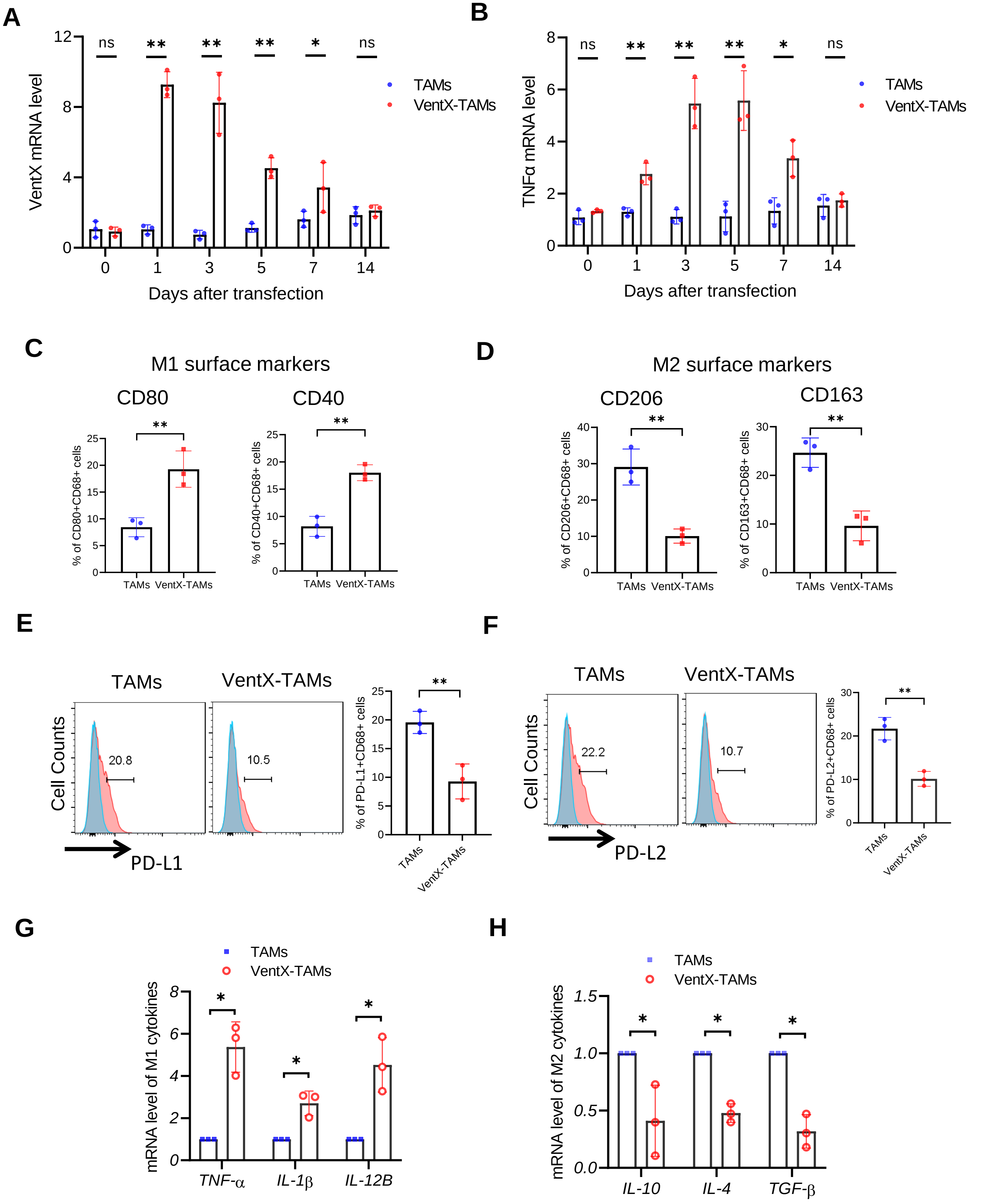
A
VentX mRNA level
ns
**
*
TAMs
VentX-TAMs
Days after transfection
B
TNFα mRNA level
C
M1 surface markers
CD80
CD40
% of CD80+CD68+ cells
% of CD40+CD68+ cells
D
M2 surface markers
CD206
CD163
% of CD206+CD68+ cells
% of CD163+CD68+ cells
E
Cell Counts
20.8
10.5
PD-L1
% of PD-L1+CD68+ cells
F
22.2
10.7
PD-L2
% of PD-L2+CD68+ cells
G
mRNA level of M1 cytokines
TNF-α
IL-1β
IL-12B
H
mRNA level of M2 cytokines
IL-10
IL-4
TGF-β

Figure S3. Effects of VentX on NSCLC-TAMs, related to Figure 3. *NSCLC-TAMs were isolated and transfected with plasmids encoding GFP or GFP-VentX. The transfected cells were cultured in RPMI 1640 medium, plus 10% FBS and 1% antibiotics for indicated days. The expressions of VentX mRNA* ***(A)*** *and TNFα mRNA* ***(B)*** *in transfected TAMs were determined by qRT-PCR analysis. After 5 days of transfection, the effects of VentX on the expression of macrophage M1 markers CD80 and CD40* ***(C),*** *M2 markers CD206 and CD163* ***(D),*** *check point inhibitor ligand PD-L1* ***(E)*** *and PD-L2* ***(F)*** *were determined by flow cytometry analysis. The effects on the expression of M1 cytokines* ***(G)*** *and M2 cytokines* ***(H)*** *were determined by qRT-PCR analysis. Data represent means ± S.D. of experiments from three NSCLC patients, "ns" stands for no statistical significant. * $p < 0.05$, ** $p < 0.05$ by paired Student's t test.*

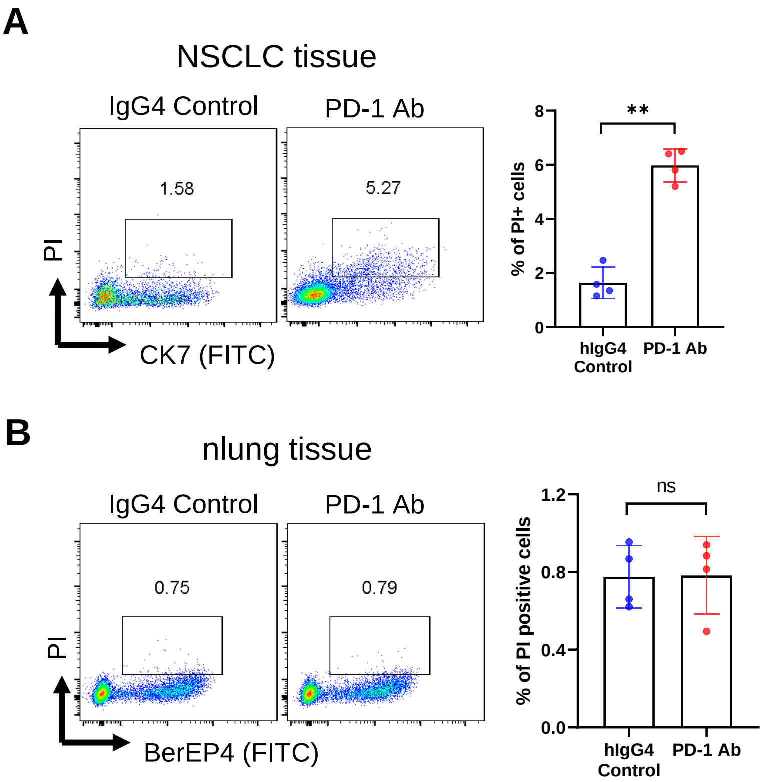

Figure S4. Tumoricidal effects of PD-1 antibody in NSCLC-TME, related to Figure 4. *En bloc NSCLS* ***(A)*** *or control nLung tissue* ***(B)*** *culture were subjected to 1 µg/mL Pembrolizumab or human IgG4 control treatment for 5 days. Single cell suspensions were then generated by mechanical disruption followed by filtering through 70 mm nylon mesh. Cancer cells from* ***(A)*** *were stained with an CK7 antibody and normal epithelial cells from* ***(B)*** *were stained with an EP4 antibody and then labeled with fluorescence dye conjugated secondary antibody. Cells were then fixed and permeabilized by fixation/permeabilization solution (Fisher Scientific), stained with PI and analyzed by a flow cytometry. Data represent means ± S.D. of three independent experiments, "ns" stands for no statistical significant. ** P <0.01 by Student t test.*

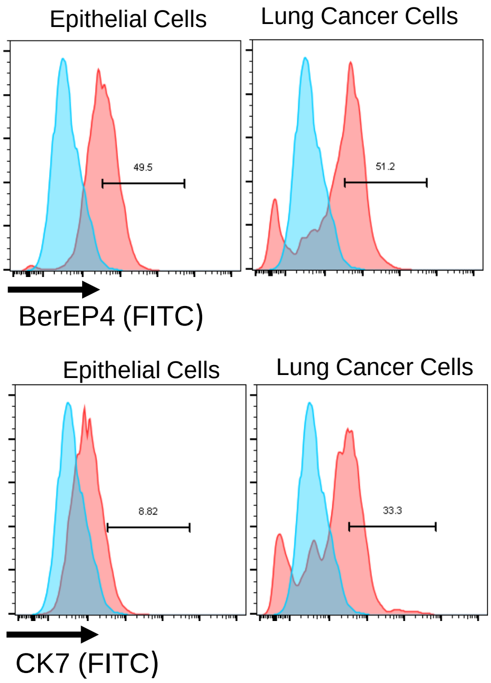

Figure S5. Surface expression of EP4 and CK7 markers in lung cancer cells and normal epithelial cells, related to Figure 5. *NSCLC and control nLung tissues were dissociated by collagenase digestion and single cell suspensions were fractioned by Ficoll-Plaque density gradient centrifugation. After the centrifugation, the layers of cancer cells and normal epithelial cells were collected and stained with CK7 and EP4 antibodies. The percentage of EP4 and CK7 positive cells in normal and cancer cells were determined by a flowcytometry.*

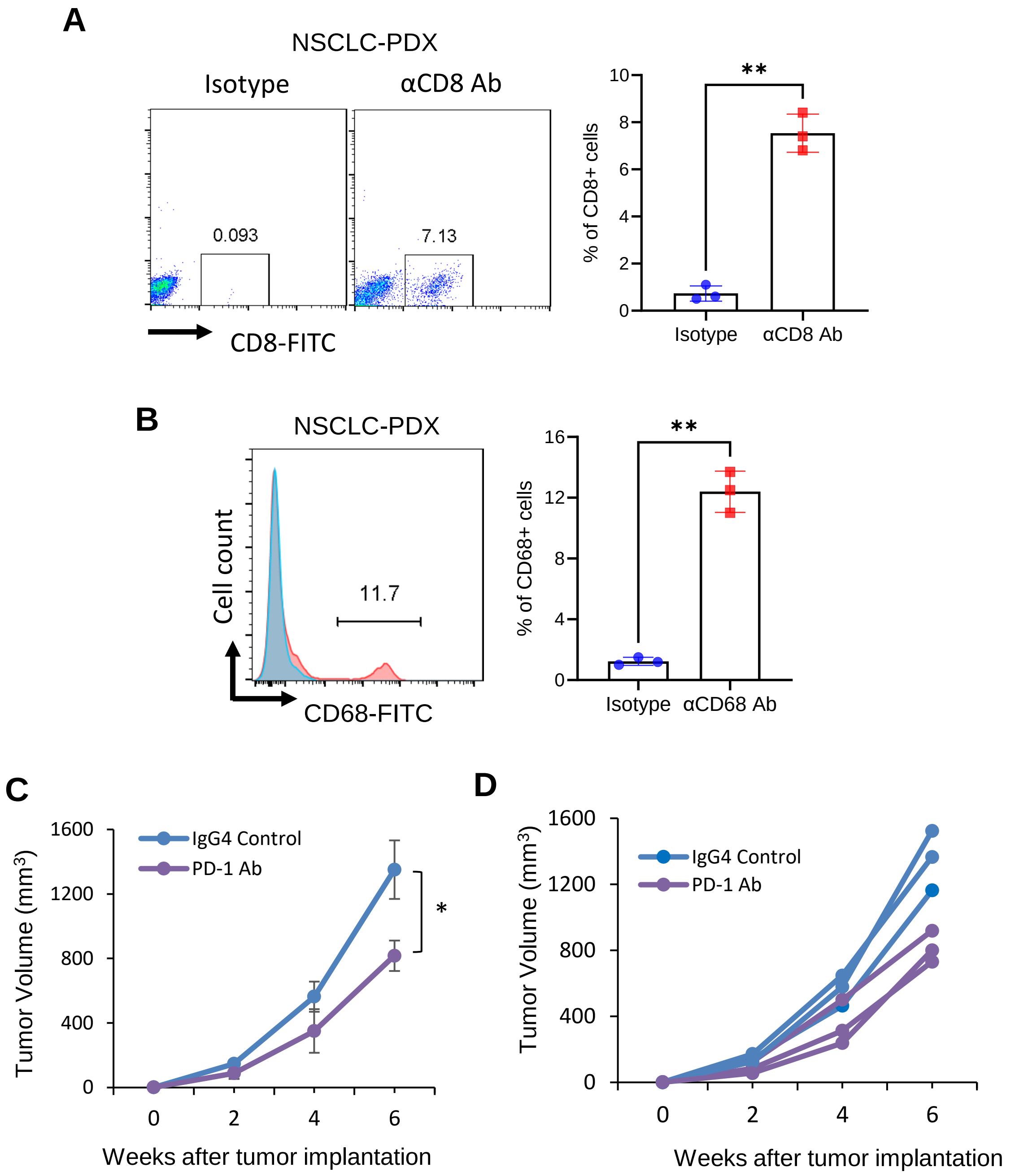

Figure S6. Effects of PD-1 antibody on tumorigenesis of NSCLC in vivo, related to Figure 6. *Individual NSG-PDX models of primary human NSCLC were generated by implantation of small pieces of NSCLC into subcutaneous space at the dorsal later side of the mice.* ***(A B)*** *Tumors from NSG-PDX-NSCLC mice were excised out and single cell suspensions were generated by mechanical disruption followed by filtering through 70µm nylon mesh. Immune cells were separated by Ficoll-Plaque density gradient centrifugation. The cells were subjected to antibody staining and the percentage of CD8+ T cells* ***(A)*** *and CD68+ macrophages* ***(B)*** *were determined by FACS analysis.* ***(C, D)*** *Growth curve of individual NSG-PDX-NSCLC treated with pembrolizumab (humanized anti-PD-1 antibody) or human IgG4 control. One week after NSCLC implantation, pembrolizumab or human IgG4 control were tail-vain injected weekly for 5 weeks. The effects of the treatment were monitored for 6 weeks. Tumor volumes were calculated according to the formula ½ (length × width^2).* ***(C)*** *Statistical analysis of treatment effects. * Indicates P<0.05, ** Indicates P<0.01 by student t-test, n=3.* ***(D)*** *Tumor growth curve in each individual NSG-PDX-NSCLC mice treated with pembrolizumab or human IgG4 control.*

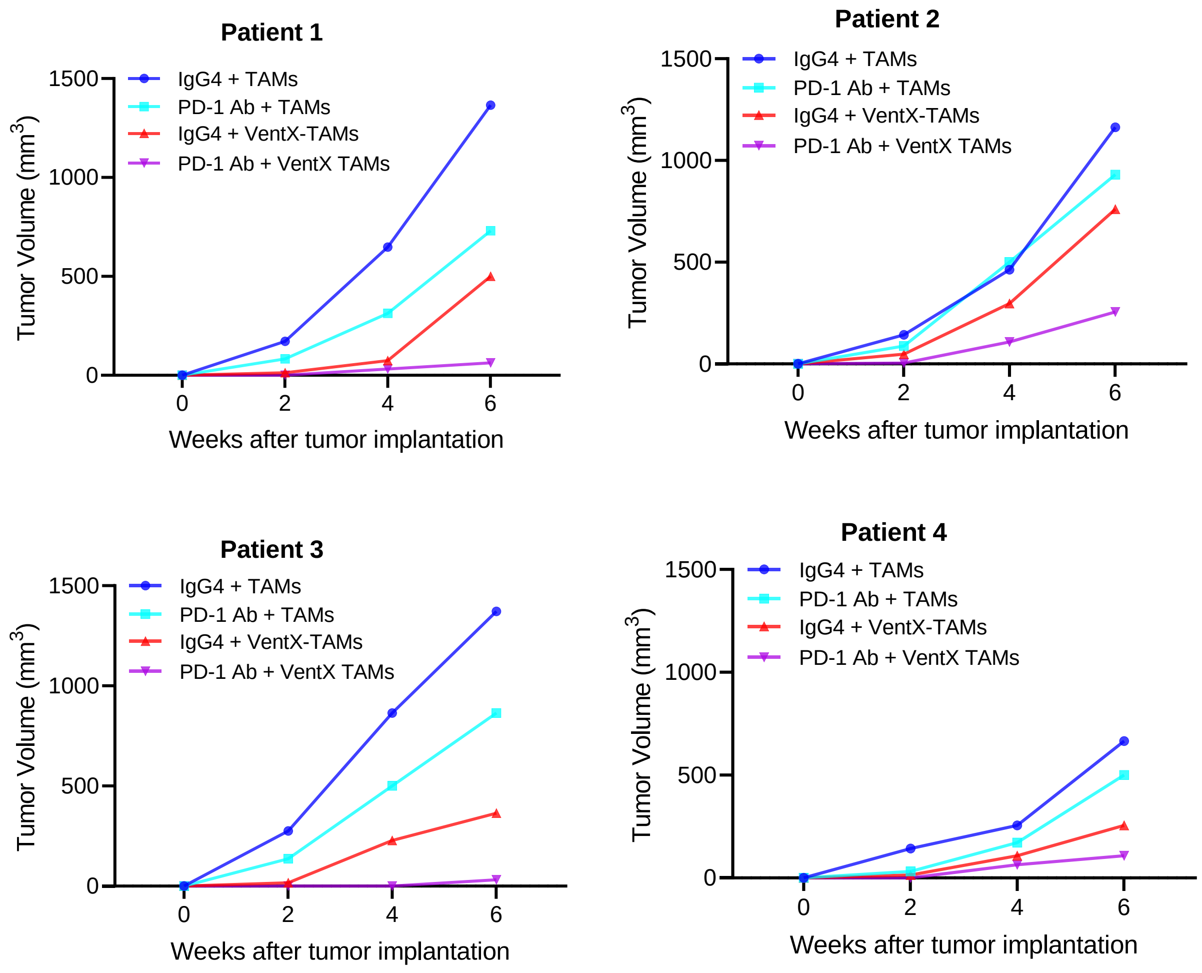

Figure S7. The effects of VentX-TAMs on PD-1 antibody treatment of NSCLC in individual patient-derived-NSG-PDX models, related to Figure 6. *Individual NSG-PDX models of primary human NSCLC derived from 4 patients were generated by implantation of small pieces of NSCLC into subcutaneous space at the dorsal later side of the mice. One week after NSCLC implantation, the mice were tail-vein injected with VentX-TAMs or control TAMs together with weekly injection of pembrolizumab or human IgG4 control for 5 weeks. The effects of the treatment on each individual tumor were monitored for 6 weeks.*

Table S1: Antibodies used in this study, related to the STAR Methods

Antibodies	Fluorophore	Clone	Source	Identifier
Anti-human CD3	APC	OKT3	eBioscience/Invitrogen	17-0037-42
Anti-human CD4	FITC	OKT4	eBioscience/Invitrogen	11-0048-42
Anti-human CD4	PE	OKT4	eBioscience/Invitrogen	12-0048-42
Anti-human CD8	FITC	SK1	Biolegend	344704
Anti-human CD8	PE	SK1	Biolegend	344706
Anti-human CD14	FITC	61D3	eBioscience/Invitrogen	11-0149-42
Anti-human CD25	PE	BC96	eBioscience/Invitrogen	12-0259-42
Anti-human CD40	PE	5C3	eBioscience/Invitrogen	12-0409-42
Anti-human CD68	FITC	Y1/82A	eBioscience/Invitrogen	11-0689-42
Anti-human CD80	PE	2D10.4	eBioscience/Invitrogen	12-0809-42
Anti-human CD152 (CTLA-4)	FITC	14D3	eBioscience/Invitrogen	11-1529-42
Anti-human CD163	PE	eBioGHI/61	eBioscience/Invitrogen	12-1639-42
Anti-human CD206	PE	19.2	eBioscience/Invitrogen	12-2069-42
Anti-human CD273 (PD-L2)	PE	MIH18	eBioscience/Invitrogen	12-5888-42
Anti-human CD274 (PD-L1)	PE	MIH1	eBioscience/Invitrogen	12-5983-42
Anti-human CD279 (PD-1)	PE	J105	eBioscience/Invitrogen	12-2799-41
Anti-human FoxP3	APC	236A/E7	eBioscience/Invitrogen	17-4777-42
Anti-human IFNγ	FITC	45.B3	eBioscience/Invitrogen	11-7319-42
Anti-hGranzyme B	PE	GB11	eBioscience/Invitrogen	12-8899-42
Anti-human IL-2	PE	MQ-17M12	eBioscience/Invitrogen	12-7029-71
Anti-human IL-17	PE	20L1509	eBioscience/Invitrogen	12-9179-42
Annexin-V	FITC		eBioscience/Invitrogen	11-8005-72
Pembrolizumab (humanized anti-PD-1 antibody, IgG4)	N/A	A2005	Selleckchem	A2005
Anti-human Cytokeratin 7 mAb	N/A	5D12	Invitrogen/Fisher	MA5-15604
Anti-human EpCAM mAb	N/A	BerEP4	Dako	M0804
FITC-Goat anti-mouse IgG (H+L)	N/A	N/A	Life Technologies	626511
Human Fc Block	N/A	2.4G2	BD Biosciences	553142

Table S2, Primer sequences used in this study, related to STAR Methods

Gene name	Forward	Reverse
VentX-C*	AAGGCAATTAGGCGCTGCTT	ACAGAACAACTGAGTCCTCCA
VentX-R*	CCGTCAGCATCAAGGAGG	CTGGACCTCTGAGAGCTGC
IL1β	AAGCTGATGGCCCTAAACAG	AGGTGCATCGTGCACATAAG
GAPDH	AGAACGGGAAGCTTGTCATC	GCCTTCTCCATGGTGGTG
IL8	ATGACTTCCAAGCTGGCCGT	CCTCTTCAAAAACTTCTCCACA
IL12B	GCAGAGGCTCTTCTGACCCCCA	AGCTGACCTCCACCTGCCGA
TNFα	CGC CAC CAC GCT CTT CTG	GCC ATT GGC CAG GAG GGC
IL4	GCTTCCCCCTCTGTTCTTCC	CTGCTCTGTGAGGCTGTTCA
IL10	GATCCAGTTTTACCTGGAGGAG	CCTGAGGGTCTTCAGGTTCTC
IL13	CCTCTACAGCCCTCAGGGAG	ATCTTGGGAATCACCCACCC
TGFB1	TACCTGAACCCGTGTTGCTCTC	GTTGCTGAGGTATCGCCAGGAA
	C*: conventional PCR; R*: real-time PCR	

Table S3, List of abbreviation and its definition used in this article, related to Figure 1 to 7

Abbreviation	Definition
CK7	cytokeratin 7
CRC	colorectal cancer
CTLA4	cytotoxic T lymphocyte associated antigen 4
CTLs	cytotoxic T lymphocytes
ICI	immune checkpoint inhibitor
IFN-γ	interferon gamma
irAE	immune-related adverse event
L-ADCA	L-adenocarcinoma
L-SCCA	L-squamous cell carcinoma
nLung	non-involved lung tissues
NSCLC	non-small cell lung cancer
NSG-PDX	NOD, scid, gamma- patient-derived xenograft
PD-1	programmed death protein 1
PDAC	pancreatic ductal adenocarcinoma
PD-L1	programmed death ligand 1
PI	propidine iodide
PTAs	pan-tumor antigens
TAM	tumor associated macrophage
TCF/LEF	T cell factor/lymphoid enhancer factor family
TGFβ	tumor growth factor beta
TME	tumor microenvironment
TIME	tumor immune microenvironment
TIME-EMS	TIME-enabling model system
TNFα	tumor necrosis factor alpha
Treg	regulatory T cells
